# Potent and Subtype-Selective Dopamine D_2_ Receptor Biased Partial Agonists Discovered via an Ugi-Based Approach

**DOI:** 10.1021/acs.jmedchem.1c00704

**Published:** 2021-06-10

**Authors:** Ana Mallo-Abreu, Irene Reyes-Resina, Jhonny Azuaje, Rafael Franco, Aitor García-Rey, Maria Majellaro, Darío Miranda-Pastoriza, Xerardo García-Mera, Willem Jespers, Hugo Gutiérrez-de-Terán, Gemma Navarro, Eddy Sotelo

**Affiliations:** † Centro Singular de Investigación en Química Biolóxica e Materiais Moleculares (CIQUS), Universidade de Santiago de Compostela, 15782 Santiago de Compostela, Spain; ‡ Departamento de Química Orgánica, Facultade de Farmacia, Universidade de Santiago de Compostela, 15782 Santiago de Compostela, Spain; § Department of Biochemistry and Physiology, Faculty of Pharmacy and Food Science, University of Barcelona, 08028 Barcelona, Spain; ∥ Faculty of Chemistry, University of Barcelona, 08028 Barcelona, Spain; ⊥ Centro de Investigación Biomédica en Red Enfermedades Neurodegenerativas (CIBERNED), 28031 Madrid, Spain; # Department of Cell and Molecular Biology, Uppsala University, Uppsala SE-75124, Sweden

## Abstract

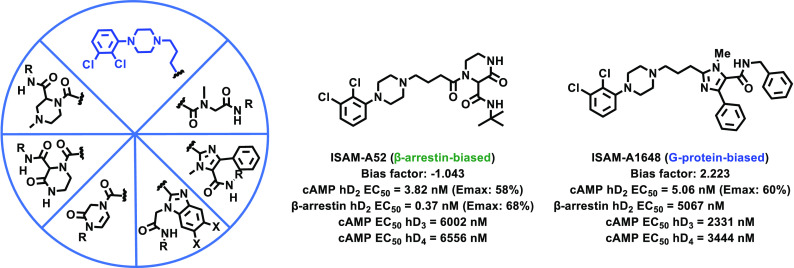

Using a previously unexplored, efficient, and versatile multicomponent method, we herein report the rapid generation of novel potent and subtype-selective DRD_2_ biased partial agonists. This strategy exemplifies the search for diverse and previously unexplored moieties for the secondary/allosteric pharmacophore of the common phenyl-piperazine scaffold. The pharmacological characterization of the new compound series led to the identification of several ligands with excellent DRD_2_ affinity and subtype selectivity and remarkable functional selectivity for either the cAMP (**22a** and **24d**) or the β-arrestin (**27a** and **29c**) signaling pathways. These results were further interpreted on the basis of molecular models of these ligands in complex with the recent DRD_2_ crystal structures, highlighting the critical role of the secondary/allosteric pharmacophore in modulating the functional selectivity profile.

## Introduction

The superfamily of seven transmembrane receptors (7TMR), commonly referred to as G protein-coupled receptors (GPCRs), is the largest target class in the druggable genome.^1^ The receptors in this superfamily regulate virtually every aspect of human physiology, and they are sensors of a wide array of extracellular stimuli.^1^ As a consequence, GPCRs are the target of more than 30% of all prescription drugs.^2^ The synergistic use of innovative experimental and computational approaches in the last decade led to the increasing appreciation of the key role of conformational plasticity on GPCR signaling events (e.g., constitutive activity, inverse agonism, or biased agonism).^3^ For a number of GPCRs, the propensity to activate distinct G proteins can elicit diverse responses depending on the cellular environment.^4^ However, an even more subtle but intriguing mode of signaling has been attributed to the ability of a receptor to activate signaling pathways independent of G-protein activation. This occurs through the scaffolding of signaling complexes by β-arrestin, a component of the GPCR desensitization and internalization machinery.^5,6^ The process by which ligands differentially modulate G-protein-dependent and/or G-protein-independent (β-arrestin) pathways to mediate specific downstream signal transduction routes is a phenomenon known as functional selectivity or biased agonism.^3^

The concept of biased agonism has progressively reshaped our understanding of GPCR signaling and shifted the paradigm of GPCR drug discovery.^7,8^ However, the molecular mechanisms behind biased signaling remain elusive since the study of the functional contributions of G-protein and β-arrestin mediated signaling pathways of endogenous/exogenous ligands still constitute a challenge.^7,9^ Biased GPCR ligands can trigger a specific pathway that is responsible for a given therapeutic effect, while not activating other signaling events eventually implicated in side effects. Such ligands are extremely useful to elucidate the key structural contributors to signal transduction pathways, besides their significant potential to develop therapeutic agents with fewer side effects.^10−12^ A paradigmatic case is Oliceridine, a G-protein-biased μ-opioid (MOR) agonist that has shown encouraging results in clinical studies, combining a potent analgesic effect with reduced incidence of β-arrestin-mediated adverse effects (e.g., respiratory depression and constipation).^13−15^

**Figure 1:**
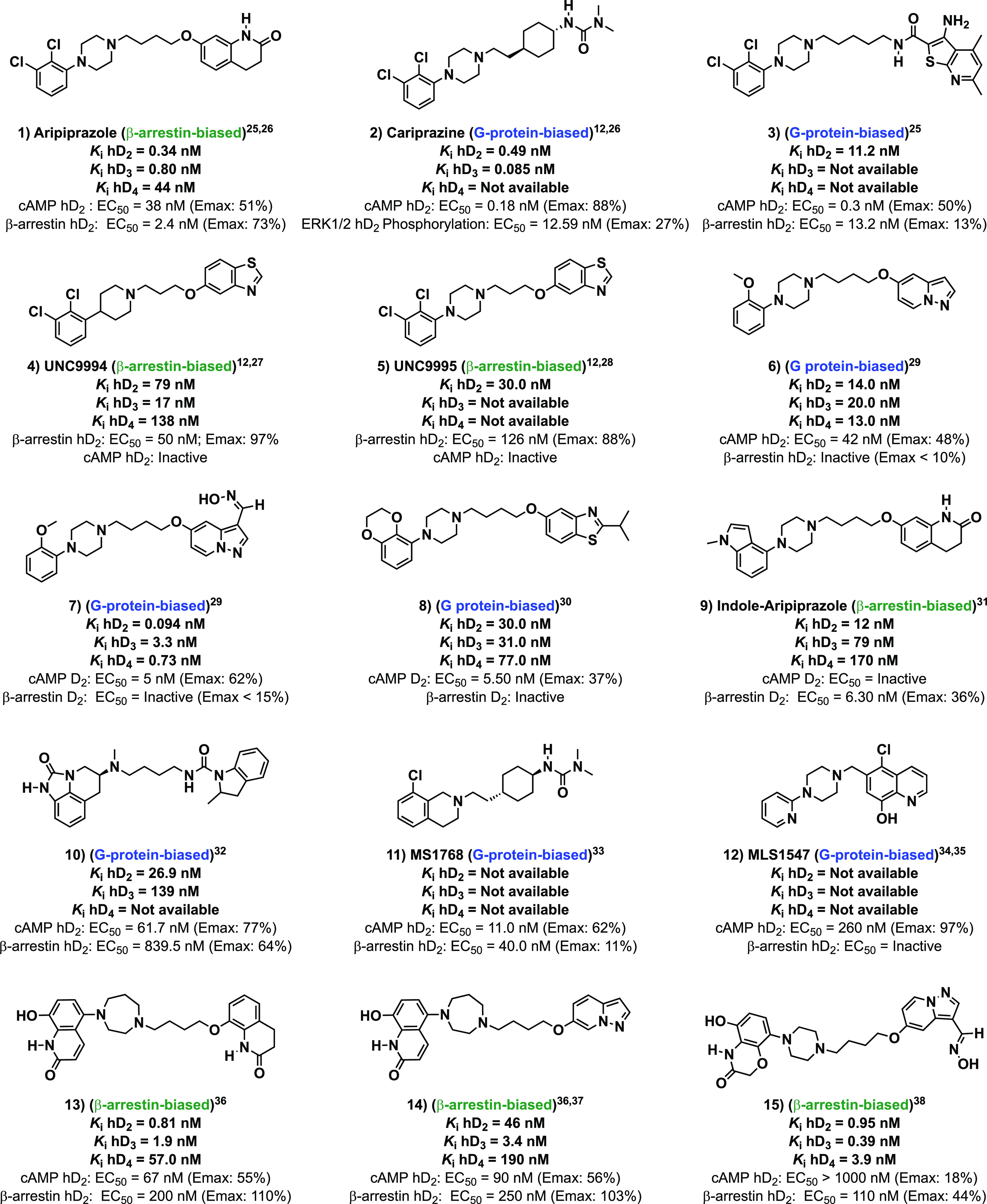
Selected examples from the literature of DRD_2_ biased ligands.^12,25−38^

The dopamine D_2_ receptor (DRD_2_) is a prototypical GPCR for which exploration of the biased agonism concept is becoming the new paradigm to provide better drugs.^12,16,17^ DRD_2_ is the primary target of antipsychotics and antiparkinsonian agents and is also implicated in the mode of action of several drugs associated with abuse and addiction.^18,19^ Schizophrenia is characterized by positive, negative, and cognitive symptoms.^20^ Classical antipsychotics are effective at targeting the positive symptoms, but they do have adverse extrapyramidal symptoms (EPS).^21^ Atypical antipsychotics have overcome some of the problems associated with typical APDs in the clinic, and they are better at targeting the positive symptoms of schizophrenia without inducing EPS. However, these compounds have their own distinct side-effect profile, which includes weight gain, agranulocytosis, and hypotension.^21^ Aripiprazole and cariprazine (Figure 1) are prototypes of a new generation of atypical antipsychotics,^22^ and they were approved by the FDA for the treatment of schizophrenia, bipolar I manic/mixed episodes, and depressive disorder.^23^ From a structural point of view, they are considered bitopic ligands, bearing a canonical primary pharmacophore (arylpiperazine) and a secondary (or allosteric) pharmacophore linked through an spacer group^24^ (Figure 1). These drugs changed the view of antipsychotic action on dopamine signaling and introduced for the first time in the treatment of psychosis a clinically relevant mechanism based on DRD_2_ occupancy without DRD_2_ blockade. They are thought to act as antagonists in the striatum, where excessive dopamine activity is believed to cause positive symptoms, but they do show agonist activity in the mesocortical pathway, where reduced dopamine activity is associated with negative symptoms and cognitive impairment.^25^

Inspired by the unique antipsychotic profile of aripiprazole, a novel series of DRD_2_ biased agonists have been developed over the past decade (Figure 1). Inspection of the pharmacological data available for these ligands enabled to identify DRD_2_ partial agonists eliciting either β-arrestin mediated recruitment and G-protein biased DRD_2_ ligands (Figure 1). A structural analysis reveals their chemical analogy, particularly with the atypical antipsychotics that inspired their design (Figure 1). While effective in retaining the desired biased profiles, the limited structural diversity inherent to their design hindered the exploration of alternative allosteric regions of the receptor (e.g., secondary pocket) adjacent to the primary (canonical) binding site. In other words, this conservative strategy could not deliver structurally novel ligands that are able to stabilize alternative conformational states of receptors.^39^ Furthermore, most DRD_2_ biased agonists developed to date do not show remarkable subtype selectivity toward the other receptors of the dopamine D_2_ family (D_3_ and D_4_, Figure 1).^40^ Evidence from clinical practice indicates that most effective antipsychotics exhibit a rather promiscuous receptor profile, with important affinity toward several GPCRs (usually defined as selectively nonselective drugs).^41,42^ However, from a chemical biology perspective, the development of molecular probes that simultaneously elicit subtype selectivity and signaling bias profiles is key to determine the molecular and physiological determinants that underpin DRD_2_ biased signaling. Such pharmacological tools would contribute to our understanding of the molecular basis of DRD_2_ signaling not only in transfected cells but also in complex and physiologically relevant environments. This information enables the elucidation of the real contribution of β-arrestin and G-protein signaling in dopaminergic receptors and the development of safer and more effective medications for schizophrenia and Parkinson’s disease.

To overcome the limitations of previous approaches, we herein report the design, synthesis, and pharmacological characterization of several series of DRD_2_ partial agonists that exhibit either G-protein or β-arrestin biased signaling profiles, uniquely combined with exquisite subtype selectivity. The new families of compounds were designed and assembled by using a highly versatile multicomponent approach. The experimental data provided structure–activity relationship (SAR) and structure–functional selectivity relationship (SFSR) trends that were consistent with the proposed binding modes, as defined in a receptor-driven docking model. The overall results of the study represent a successful proof-of-concept of an unexplored strategy for the rapid identification of novel structurally diverse and functionally selective DRD_2_ ligands.

## Results and Discussions

### Design and Synthesis

**Figure 2:**
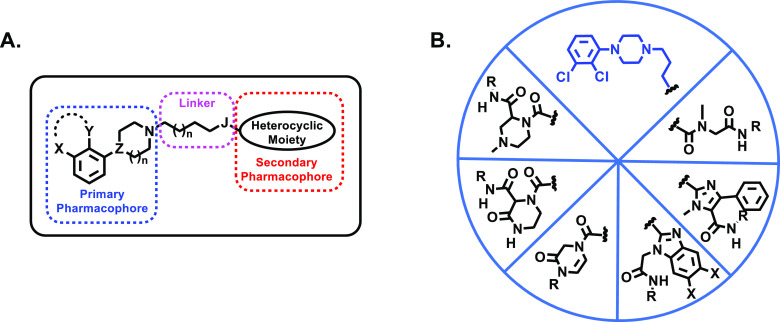
(A) Representative structure of most DRD_2_ biased ligands. (B) General structure of herein described ligands. Blue: common (primary) pharmacophore in the series. Black: structure of the scaffolds explored in the secondary pharmacophore.

**Scheme 1:**
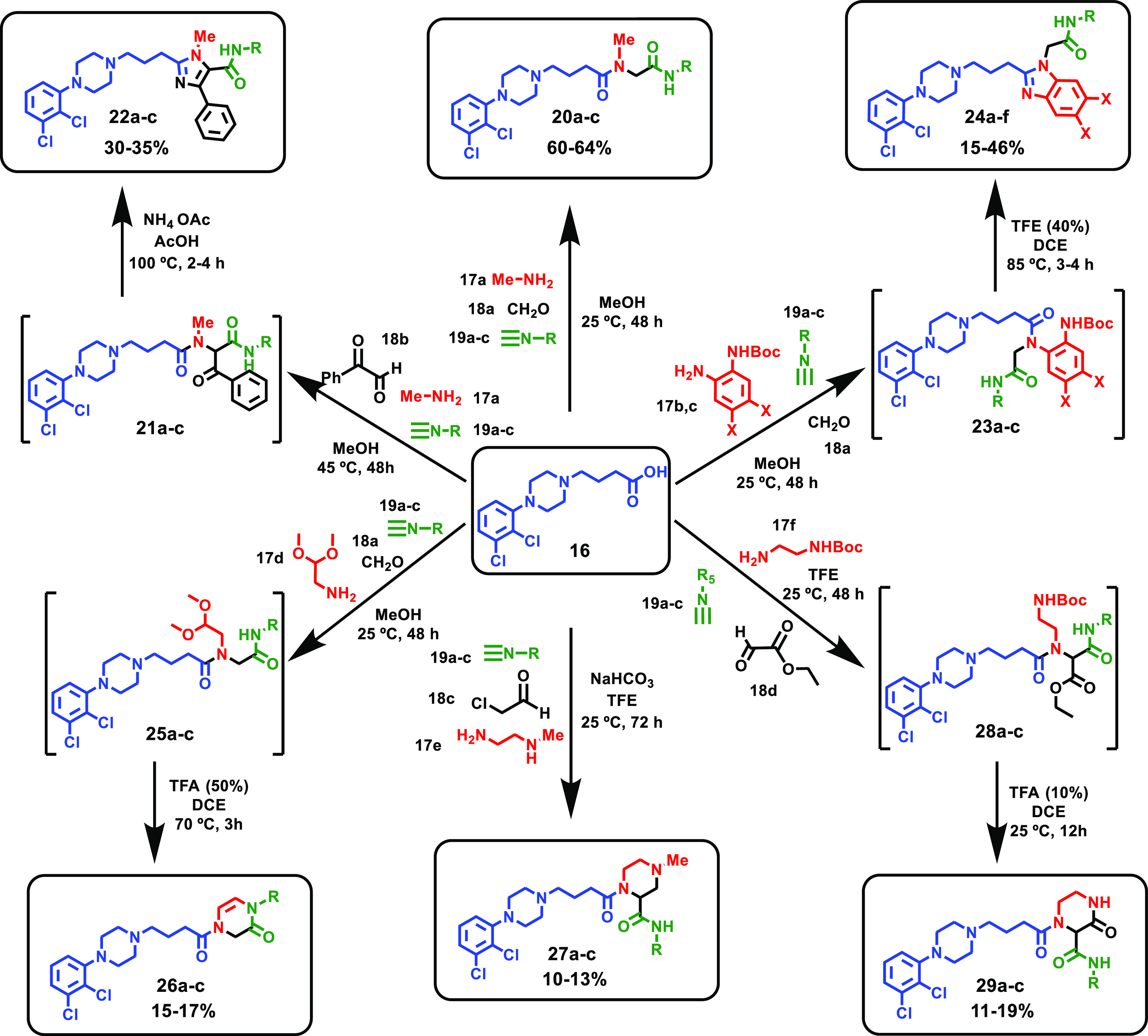
Ugi-Based Assembly of the Target Compounds

From a structural point of view, most studied DRD_2_ biased ligands fit in the pharmacophoric model presented in Figure 2. These compounds are bitopic ligands, containing three well-defined regions:^43^ (1) the primary pharmacophore (PP) [commonly referred to as the left-hand side (LHS) or head group], consisting of a mono- or disubstituted phenyl-piperazine scaffold, (2) the central linker, that is usually variable in length and nature (e.g., acyclic or cyclic), and (3) the secondary (or allosteric) pharmacophore (SP) [commonly referred to as the right-hand side (RHS) or tail group], generally consisting of a heterocyclic core. Although the aromatic piperazines of the primary pharmacophore (PP) dictate the efficacy profile and is sufficient to allow binding to the primary (orthosteric) binding site of DRD_2_ (and to that of the closely related DRD_3_ subtype), enlargement of the chemical structures by addition of a flexible linker and a second, mostly lipophilic system (SP) has been found to promote enhanced affinity and subtype selectivity.^44,45^ In the present study, it was decided to maintain the primary pharmacophore (Figure 2), with the 1-(2,3-dichlorophenyl)piperazine moiety selected (which is present in aripiprazole), and a shorter than usual (four atoms) linear linker, which is present in UNC9995^12,33^ (Figure 1). Six previously unexplored secondary pharmacophoric (SP) groups (Figure 2) were proposed to examine the effect of these structural modifications on subtype selectivity (DRD_2_, DRD_3_, and DRD_4_) and also their effect on the DRD_2_ functional selectivity profile of the novel ligands. The selected SP frameworks provide novel topologies, physicochemical features, and alternative binding modes that should enable the capture of diverse conformational states within the receptor. In addition to the heterocyclic and functional diversity introduced, some of the proposed SP fragments bear a stereogenic center within the heterocyclic framework (Scheme 1, compounds **27** and **29**), thus introducing stereochemical diversity that would enable the future investigation of scarcely explored stereoselective interactions within the SP region.

The appropriate decoration of targeted structures (Figure 2), according to the specific requirements of the target receptor, would allow effective interaction with subpockets in the secondary (allosteric) binding site, ultimately resulting in optimized bioactivity levels. Moreover, the achievement of such decorations by synthetically feasible approaches is an emerging criterion for library design. The implementation of concise and efficient synthetic methodologies that reconcile molecular complexity with experimental simplicity, thus allowing rapid access to privileged molecular frameworks, constitutes a highly desirable goal within the competitive environment of drug discovery. A valuable addition to the compendium of preparative methods to generate drug candidate libraries is provided by multicomponent reactions (MCRs),^46,47^ which have emerged as a tailored synthetic paradigm in the context of medicinal chemistry and chemical biology programs. MCRs combine three major principles in organic synthesis: convergence and atom and step economies. In addition, such reactions are highly flexible and their extraordinary exploratory power allows maximum structural complexity to be generated from simple starting materials in just a single step.^48^ The main goal of the study reported here was to identify novel DRD_2_ biased agonists by introducing unexplored structural elements in the secondary pharmacophore (SP) region. The selection criteria for the proposed SP groups were guided by the principles of synthetic feasibility and structural diversity (Figure 2). Thus, we envisioned different divergent, highly exploratory, and experimentally simple MCR-assisted pathways (Scheme 1). The selected synthetic approaches, which are based on the Ugi four-component reaction (U-4CR), exploit the potential of this transformation for structural diversification. Thus, starting from the readily available carboxylic acid **16**, which contains the primary pharmacophoric moiety and the linker, we envisioned a set of reactions (Scheme 1) in which **16** would be combined with diversely functionalized amine inputs (**17**), carbonyl partners (**18**), and three representative isocyanides (**19**).

The simplest set of ligands (**20a**–**c**) was obtained by the Ugi reaction of **16** with methylamine (**17a**), formaldehyde (**18a**), and isocyanides **19a**–**c** in methanol at room temperature for 48 h.^47^ The assembly of the other five subsets (**22**, **24**, **26**, **27**, and **29**) involved the use of polyfunctional reactive substrates and/or the versatility of the Ugi-Deprotect-Cyclize (UDC) strategy.^47,49,50^ As shown in Scheme 1, the feasibility of the selected pathways relies heavily on the latent reactivity of the different functionalized Ugi adducts (**21**, **23**, **25**, and **28**), which, upon direct cyclization (**22**, **26**, and **27**) or removal of the protecting group, undergo an intramolecular cyclization (**24** and **29**) to furnish the target structures in an efficient transformation that takes place in one pot. In this way, compounds **22a**–**c** were obtained by reaction of **16** with methylamine (**17a**), isocyanides **19a**–**c**, and phenylglyoxal (**18b**) as the key precursor (Scheme 1). The superior reactivity of the formyl group in **18b** ensured the chemoselectivity of the reaction to produce an Ugi adduct (**21a**–**c**) that contains an enolizable ketone group, and this was transformed *in situ* to give **22a**–**c** by treatment with ammonium acetate at 100 °C.^51^ Treatment of the carboxylic acid **16** with formaldehyde (**18a**), isocyanides **19a**–**c**, and the mono-BOC protected phenylenediamines **17b**–**c** afforded the Ugi adducts **23**, which, upon acidic BOC cleavage and thermal treatment, afforded **24a**–**f** (Scheme 1).^52^ The Ugi reaction of **16**, formaldehyde (**18a**), isocyanides **19a**–**c**, and aminoacetaldehyde dimethyl acetal (**17d**) generated the adducts **25**, which were transformed *in situ* to **26a**–**c** by an acid-mediated transformation that involved an intramolecular cyclization and subsequent elimination (Scheme 1).^53^

The four-component reaction of **16**, isocyanides **19a**–**c**, and the bifunctional precursors **18c** (chloroacetaldehyde) and **17e** (*N*-methylethylenediamine) under basic conditions (NaHCO_3_) directly afforded the piperazine derivatives **27a**–**c** (Scheme 1).^54^ The sequence involves the formation of an Ugi adduct, which, under basic conditions, undergoes an intramolecular nucleophilic substitution reaction. Finally, the assembly of piperazin-2-ones **29a**–**c** was accomplished by a similar pathway (Scheme 1), starting from carboxylic acid **16** and isocyanides **19a**–**c**, but using two alternative bifunctional precursors [i.e., ethyl glyoxylate (**18d**) and *N*-BOC-ethylenediamine (**17f**)]. Acid-mediated cleavage of the BOC group in the Ugi adducts **28** provided the target ligands (**29**).^55^ Ligands of series **27** and **29**, which contain a stereocenter within the SP heterocyclic fragment, were isolated and evaluated as racemic mixtures. A detailed description of the synthetic methods and the complete structural, spectroscopic, and analytical data for all compounds are provided in the Experimental Section.

The five heterocyclic cores explored as secondary pharmacophoric groups (**22**, **24**, **26**, **27**, and **29**) can be considered as conformationally restricted analogs of the early acyclic series **20**, with differences in structure, topology, physicochemical descriptors, and complexity. Thus, the Ugi-based diversification strategy enables the rapid differentiation of the structural elements of the acyl-aminoamide scaffold into highly diverse molecular frameworks.

### Biological Evaluation

The newly synthesized ligands were all initially tested in cAMP inhibition assays with three dopamine receptor subtypes (DRD_2_, DRD_3_, and DRD_4_), i.e., the DRD_2_-like receptors, to evaluate their functional behavior and selectivity profile (Table 1). All experiments were performed *in vitro* on transfected HEK-293T cells, with the evaluation of the efficacy (E*_max_*) and half maximum inhibitory concentration (IC_50_) for the cAMP assays, using previously described experimental protocols.^56^ Quinpirole was used as a control and reference drug during these studies. Compounds **27a–c** and **29a–c** were tested as racemic mixtures.

**Table 1:**
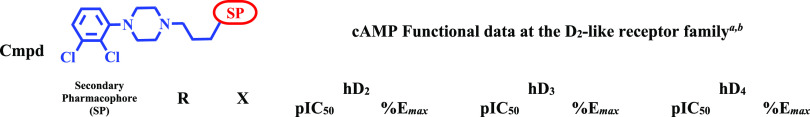

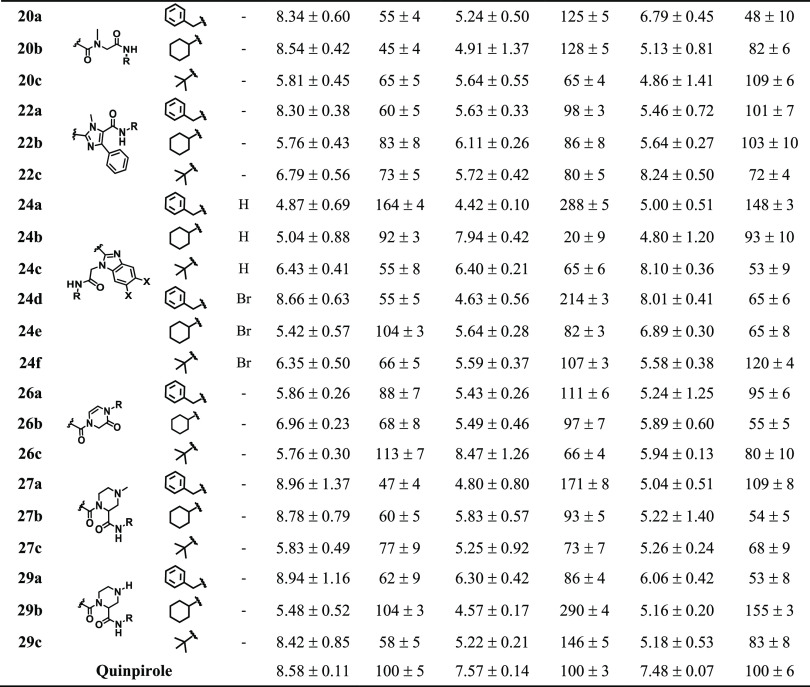
Structures and Pharmacological Data at the D_2_ Receptor Family for the Ligands^a^*^,^*^b^

a pIC_50_ and E*_max_* values are the average of five experiments, each performed in duplicate with ± SEM values that are three times lower than the average. E*_max_* relative to the effect of the reference agonist quinpirole.

b Tested using the experimental protocols described in the Experimental Section.

On the basis of its DRD_2_ potency (pIC_50_ > 8) and subtype selectivity criteria, seven ligands (**20a**, **20b**, **22a**, **27a**, **27b**, **29a**, and **29c**) were selected for further investigation of the DRD_2_-mediated potency (EC_50_) and efficacy (E*_max_*) for β-arrestin-2 recruitment (Table 2). As a consequence of its excellent DRD_2_ potency (pIC_50_ = 8.66), albeit without selectivity toward DRD_4_, ligand **24d** was also included in the set of compounds selected for bias characterization. The β-arrestin-2 recruitment study involved BRET experiments performed on transfected HEK-293T cells using previously described experimental protocols.^57^ Aripiprazole and quinpirole were employed as controls during these studies.

**Table 2:**
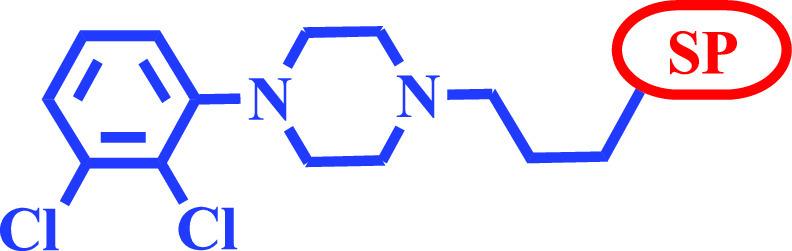

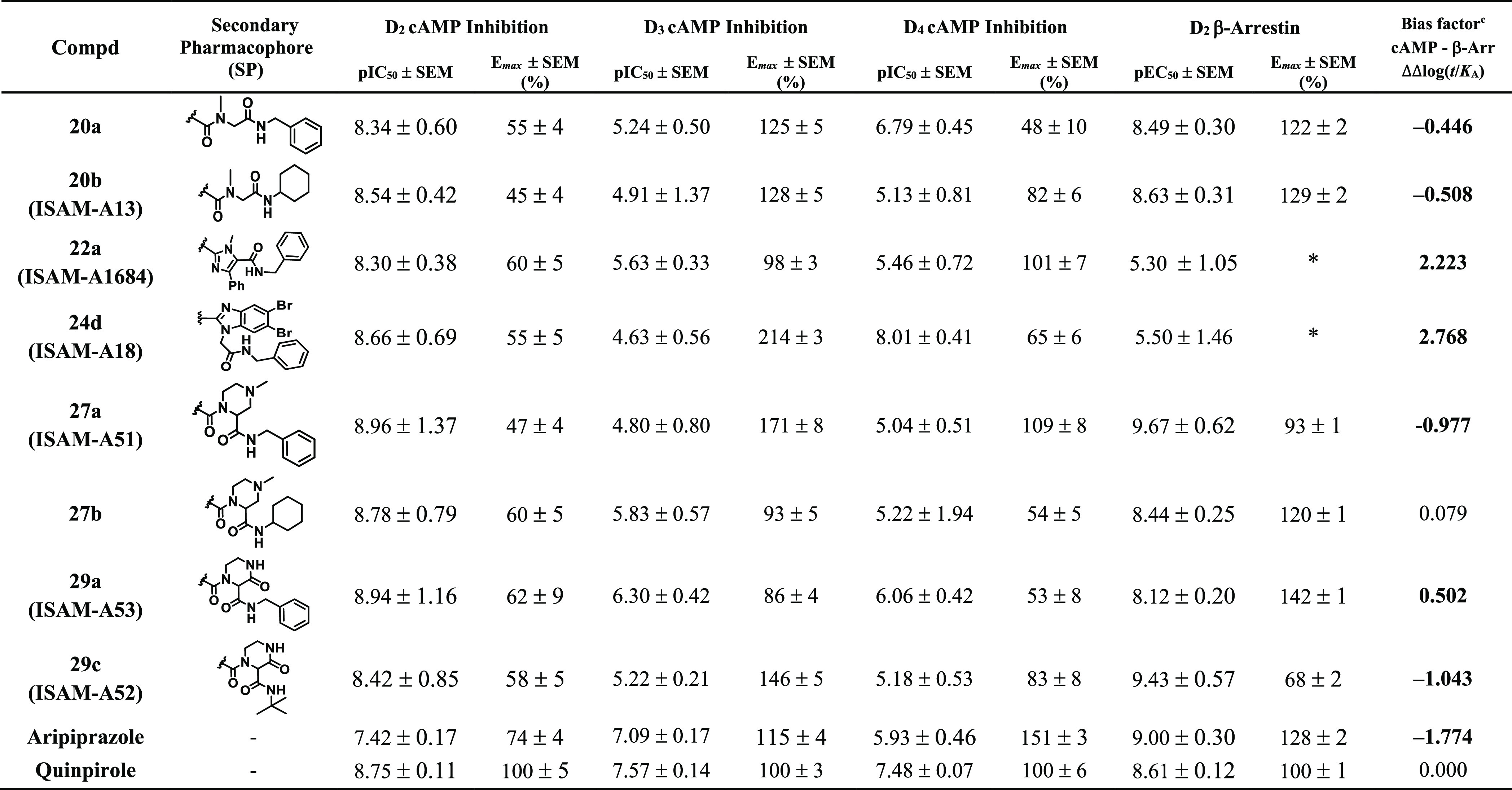
Ligands and Structure–Selectivity Relationship (SSR) Data for Selected Ligands^a^*^,^*^b^

a EC_50_, IC_50_, and E*_max_* values are the average of five experiments, each performed in duplicate with ± SEM values that are 3-fold less than the average. E*_max_* relative to the effect of the reference agonist quinpirole.

b Tested using the experimental protocols described in the Experimental Section.

c Bias factors were quantified by the operational model using quinpirole as a positive control (see the Experimental Section). Ligand bias values >0 indicate preference for the cAMP pathway, and values < 0 indicate preference for the β-arrestin signaling pathway. Values above 0.5 are considered significant and are highlighted. *E*_max_* is not shown due to low affinity of the ligand.

**Figure 3:**
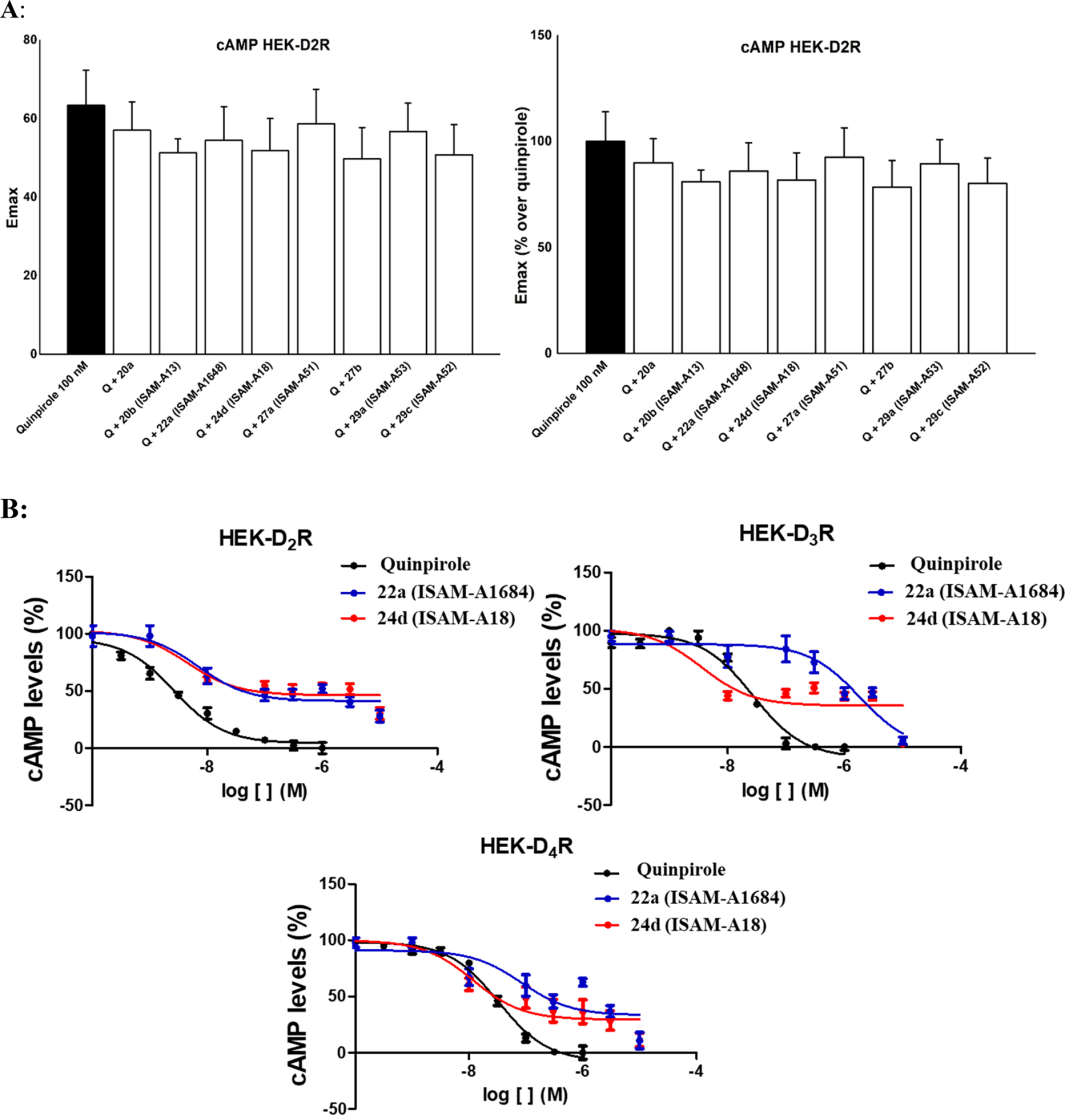
(A) E*_max_* values for 100 nM quinpirole in cAMP assays performed in HEK-293T cells expressing DRD_2_, pretreated or not (reference black column) with 100 nM of the selected compounds. Data are normalized (right) to the effect of quinpirole alone (100%). (B) HEK-293T cells expressing human DRD_2_, DRD_3_, or DRD_4_ were treated with the indicated compounds. The effect of the compounds on the decrease of 500 nM-induced cAMP levels was determined as described in the Experimental Section. Data are given relative to the value of forskolin alone and then normalized to the effect of quinpirole.

With the aim of better exploring the pharmacological profile of the novel series herein documented it was decided to evaluate a set of ligands (Table 2, **20a**, **20b**, **22a**, **24d**, **27a**, **27b**, **29a**, and **29c**) in antagonist mode. In this case, cells were pretreated with the selected compounds before treatment with the agonist quinpirole (see Methods). As can be observed in Figure 3A, there were no significant variations in the efficacy between each of the compounds tested with quinpirole compared to the quinpirole tested alone. These results enable to discard a potential antagonistic behavior for the studied compounds. Figure 3B shows a comparative profile of the cAMP dose response curves obtained for ligands **22a** and **24d** and quinpirole at DRD_2_, DRD_3_, and DRD_4_.

### Structure–Activity and Structure–Selectivity Relationships

The cAMP functional data for the novel compounds (Table 1) reveal that some ligands behave as DRD_2_ selective partial agonists. Inspection of reported data enables to identify eight novel and highly potent (pIC_50_ > 8) DRD_2_ ligands (e.g., **20a**, **20b**, **22a**, **24d**, **27a**, **27b**, **29a**, and **29c**), six of which elicit remarkable selectivity (>1000-fold) toward DRD_3_ and DRD_4_. Furthermore, some potent and selective DRD_3_ (i.e., **24b** and **26c**, pIC_50_ = 7.94 and 8.47, respectively) or DRD_4_ (i.e., **22c** and **24c**, pIC_50_ = 8.24 and 8.10, respectively) ligands were identified. These data emphasize the potential of herein disclosed MCR-based diversification of the secondary pharmacophore region has in modulating the interaction with DRD_2_. Additionally, our results exemplify how subtle structural modifications on the secondary pocket can provoke important differentiation in the biological profile of the synthesized ligands.

For a more immediate and efficient analysis of the variation of both affinity and selectivity, the pIC_50_ values at DRD_2_ (*X* axis) versus DRD_3_ (*Y* axis, top panel) and DRD_4_ (*Y* axis, bottom panel) are provided as independent scatter plots using the same scale and range for both axes (square plot). Each subset was represented in a different color and shape in order to facilitate a more comprehensive analysis of both potency and selectivity within a series. In both plots, the DRD_2_ selective compounds appear below the diagonal (right bottom zone), with the distance from the diagonal being proportional to the degree of selectivity, confirming that the identified DRD_2_ partial agonists also show a high degree of selectivity versus DRD_3_/DRD_4_. This subset was selected for further pharmacological characterization (see Table 2).

The functional data presented in Table 1 highlight the relevance of the amide group in the secondary pharmacophore for effective interaction within DRD_2_. The only subset that did not provide potent DRD_2_ ligands (**26**) has this amide group embedded within the heterocyclic core, which means that they lacked the polar hydrogen and had a conformational restraint, while the rest of the series provided at least one ligand with significant DRD_2_ potency. In contrast to the low affinity on the DRD_2_, series **26** provided compound **26c**, a highly potent (pIC_50_ = 8.47) and selective (>300-fold) novel DRD_3_ partial agonist.

**Figure 4:**
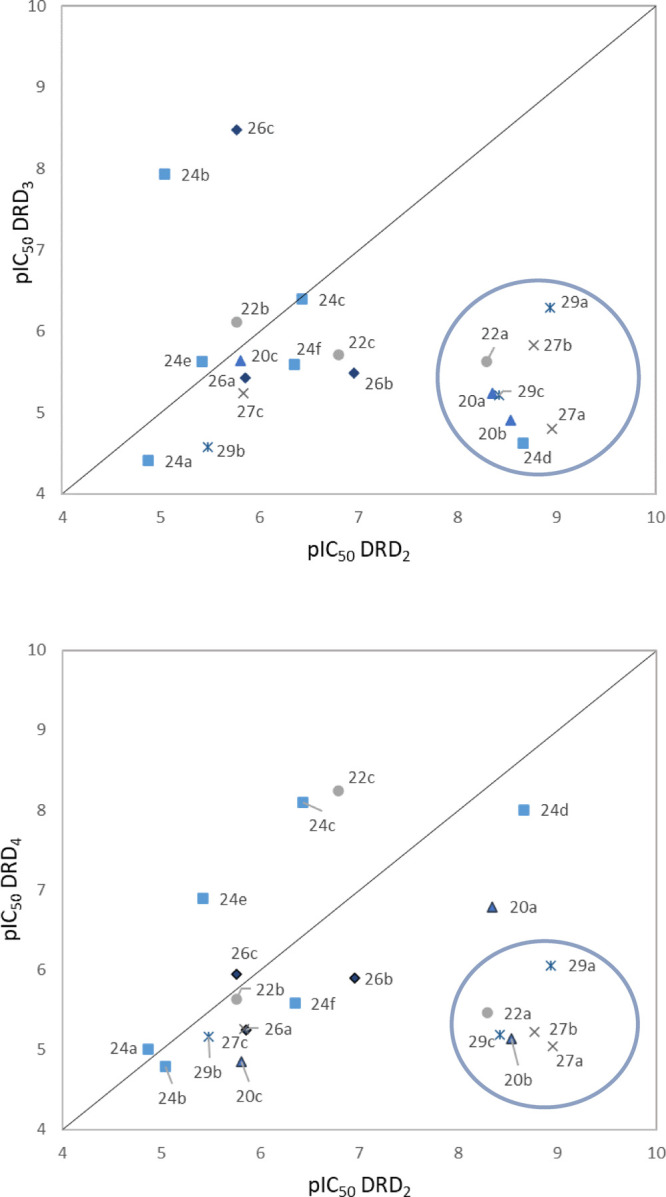
Potency–selectivity DRD_3_–DRD_2_ and DRD_4_–DRD_2_ plots.

Series **20**, **27**, and **29** generally yielded potent and subtype-selective DRD_2_ partial agonists, and these included the most attractive ligands identified in this study (Table 1 and Figure 4). In these series, compounds bearing a benzyl group on the amide moiety (**20a**, **27a**, and **29a**) systematically exhibited a low nanomolar potency (pIC_50_ = 8.34, 8.96, and 8.94 respectively). In contrast, the cyclohexyl group seems to be well tolerated only in acyl-aminoamides (**20b**) and the *N*-methylpiperazines (**27b)**. Conversely, those compounds that contained a *tert*-butyl residue generated ligands (**20c** and **27c**) that systematically exhibited micromolar potency, apart from **29c**, thus suggesting that this group could not facilitate optimal complementarity within DRD_2_. Although most ligands with imidazole- or benzimidazole-based SP groups (Table 1, ligands **22** and **24**) have low potency at DRD_2_, the pIC_50_ values determined for ligands **22a** and **24d** (pIC_50_ = 8.30 and 8.66, respectively) reveal that these scaffolds, when appropriately decorated on the exocyclic amide group (i.e., with a benzyl group), can provide potent and selective DRD_2_ partial agonists.

As previously discussed, 1-acyl-*N*-methylpiperazine-2-carboxamides **27** and 1-acyl-*N*-methyl-3-oxopiperazine-2-carboxamides **29** can be considered as conformationally restricted analogs of the acyl-aminoamides **20**. Thus, their similar biological profile (potency and selectivity) could be a consequence of the close structural similarity of these three series. Despite the structural analogy, the cyclic constrained analogs (piperazine-2-carboxamides **27** and 3-oxopiperazine-2-carboxamides **29**) exhibited slightly superior potency (Table 1) when compared to the acyclic series (**20**). This trend suggests that the cyclic derivatives are more similar to the bioactive conformation. As observed in the early series, and with the exception of **29c** (pIC_50_ = 8.42), ligands bearing the *tert*-butyl group in the exocyclic amide afforded the weakest potency (pIC_50_ = 5.76–6.79). Another interesting structural feature of the conformationally restricted series **27** and **29** is the presence of a stereogenic center at position 2 of the heterocyclic core. Although these compounds were tested as racemates, it is reasonable to expect diverse pharmacological profiles for the different enantiomers. Accordingly, the potential influence of the absolute configuration of the stereogenic center in these series will be explored in future work.

DRD_2_-mediated signaling events are initiated either by G protein-dependent (G-protein-coupled) and/or G protein-independent pathways (β-arrestin recruitment). The ability of a (partial) agonist to selectively activate one of these specific signaling pathways is a pharmacological phenomenon known as functional bias (or functional selectivity). A key goal of this study was to explore the relationship between the biased selectivity and the structural features of the ligand, which ultimately lead to the establishment of specific interactions with DRD_2_. Thus, we selected the seven derivatives (**20a**, **20b**, **22a**, **27a**, **27b**, **29a**, and **29c**) that exhibited a high cAMP potency (pIC_50_ > 8) and optimal DRD_2_ selectivity (Table 1) to perform a β-arrestin-2 recruitment BRET assay in transfected HEK-293T cells, which determines the potency and efficacy for β-arrestin-2 recruitment. Although it was not selective (DRD_4_, pIC_50_ = 8.01), the benzimidazole derivative **24d** was included in this study due to its excellent DRD_2_ potency (pIC_50_ = 8.66). Aripiprazole, a known biased ligand, was used as a reference ligand and quinpirole^58^ (a full agonist of DRD_2_) was used as a positive control in both cAMP and β-arrestin-2 recruitment BRET assays. The comparative cAMP and β-arrestin-2 data are presented in Table 2. In order to identify functional bias rapidly, a bias factor was calculated using the Black and Leff operational model^59^ with respect to quinpirole (see Table 2). Most of the evaluated ligands exhibited excellent efficacy in the β-arrestin recruitment pathway (E*_max_* over quinpirole in the range 68–142%, see Table 2), thus behaving as full agonists for this pathway. The most salient data emerging from β-arrestin recruitment assays evidenced two pairs of ligands that elicit opposite signaling profiles. Thus, while ligands **27a** and **29c** exhibit a very attractive sub-nanomolar profile in the β-arrestin recruitment test (pIC_50_ = 9.67 and 9.43, respectively), derivatives **22a** and **24d** showed only weak potency (micromolar range). The availability of ligands bearing different groups on the exocyclic amide in series **20**, **27**, and **29** provided evidence of the key role of the alkyl group (benzyl, cyclohexyl, or *tert*-butyl) on the β-arrestin recruitment potency. Interestingly, the compounds that elicited the poorest β-arrestin recruitment potency (**22a** and **24d**) contain an aromatic heterocyclic core with an *N*-benzyl group within the secondary pharmacophore.

Six of the ligands (**20b**, **22a**, **24d**, **27a**, **29a**, and **29c**) showed a clear functional selectivity profile (biased agonism) according to the bias factor parameter (Table 2), where a positive value indicates a preference for the cAMP pathway and a negative value denotes that β-arrestin recruitment is dominant. As one would expect, the weak potency in the β-arrestin recruitment assay and excellent cAMP data mean that ligands **22a** and **24d** show a significant bias toward cAMP [ΔΔlog(τ/K_A_) = 2.223 and ΔΔlog(τ/K_A_) = 2.768, respectively]. These values represent 167-fold and 586-fold bias, respectively, toward the cAMP pathway. Furthermore, compound **29a** also shows a moderate [ΔΔlog(τ/K_A_) = 0.502] 3-fold bias toward cAMP inhibition. In contrast, ligands **27a** and **29c**, due to their sub-nanomolar effect and excellent efficacy in the β-arrestin pathway (pEC_50_ = 9.67 and 9.43, respectively) and its potency and moderate efficacy in the cAMP pathway, showed 10-fold and 11-fold β-arrestin biased agonism. Compound **24d**, besides being one of the most potent binders at DRD_2_ and indeed the partial agonist with strongest bias toward the cAMP pathway (Table 2), lacks the required D_2_/D_4_ selectivity profile (Table 1) to warrant further characterization of this particular compound. In any case, compound **24d** was used as a tool to understand the molecular basis of its biased profile.

**Figure 5:**
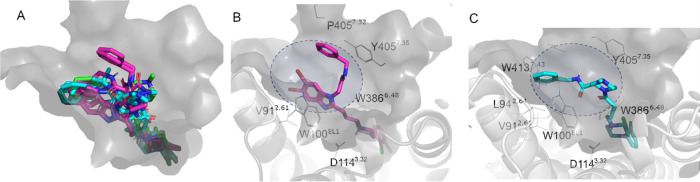
Binding mode of compounds in Table 2 on the inactive DRD_2_, PDB code 6CM4 (A). Compounds are color coded according to their pharmacological activity as G-protein biased (magenta), β-arrestin biased (cyan), or no bias (green). The detailed binding mode for each compound class is shown, for compound **24d** (B) and compound **27a** (C), depicting the residues involved in interactions with the ligand in each case, and the variable region occupied by the SP designated with a blue circle.

In order to investigate the structural basis for the different biased signaling profiles, a complex of each of the molecules listed in Table 2 with DRD_2_ was generated by different docking approaches, initially using the crystal structure of DRD_2_ in complex with risperidone.^60^ Despite the fact that this is an inactive conformation of the receptor, the chemical similarity of the general scaffold of our compounds with the co-crystallized antagonist (risperidone) supported the use of this crystal structure. Moreover, the orthosteric binding site of the aminergic receptor is not expected to change substantially upon complexation with partial agonists.^61^ As derived from the binding mode obtained (Figure 5), the 2,3-dichlorophenyl ring on the piperazine scaffold (primary pharmacophore, commonly referred to as LHS) is analogous to the benzisoxazole moiety of risperidone, which uniquely extends into a deep binding pocket defined by the side chains of residues in TM3 (Cys118^3.36^ and Ile122^3.40^), TM5 (Ser197^5.46^ and Phe198^5.47^), and TM6 (Phe382^6.44^, Phe390^6.52^, and Trp386^6.48^) as opposed to other dopaminergic ligands crystallized to date.^60,62,63^ The common anchoring point throughout the series is the salt bridge interaction between the charged nitrogen in the piperazine and the sidechain of Asp114^3.32^. The position of the secondary pharmacophore (SP) is, as expected, more variable. Interestingly, there is a correlation between the pharmacological activity and the structural features introduced, thus providing an initial proposal for the structural interpretation of ligand bias on this receptor. Thus, the two strongest G-protein biased ligands (**22a** and **24d**, magenta in Figure 5) place the benzyl tail toward the extracellular region, thus making distinct contacts with the tip of TM7 (Pro405^7.32^ and Tyr408^7.35^). This arrangement is in contrast to the other benzyl-containing SP, which were moderately selective for the β-arrestin signaling pathway (**20a** and **27a**) and bend the SP toward TM2 (Val91^2.61^ and Leu94^2.64^, ligands in cyan in Figure 5). A similar orientation was found for the cyclohexyl substituent in ligands **20b** (β-arrestin-biased) and **27b** (non-biased) or even for the benzyl-containing **29a**, which shows a less pronounced bias toward the G-protein pathway. According to this model, imidazole- or benzimidazole-based SP groups, specifically decorated with the exocyclic amide benzyl substituted, occupy a distinct subpocket that might be related to their G-protein biased profile, while there is no clear specific subpocket for β-arresting biased ligands. Additionally, this binding mode agrees with the previous observations of the so-called extended binding domain (EBD) playing a role in the DR subtype specificity.^60,62,63^ The recent crystal structure of DRD_2_ with the atypical antipsychotic haloperidol^64^ demonstrated the flexible nature of this pocket, where the conserved Trp100 in EL1 can open a new space for the rigid substituent of this molecule, thus opening the door to a more comprehensive dynamic characterization of the binding mode of different DRD_2_ ligands.

**Figure 6:**
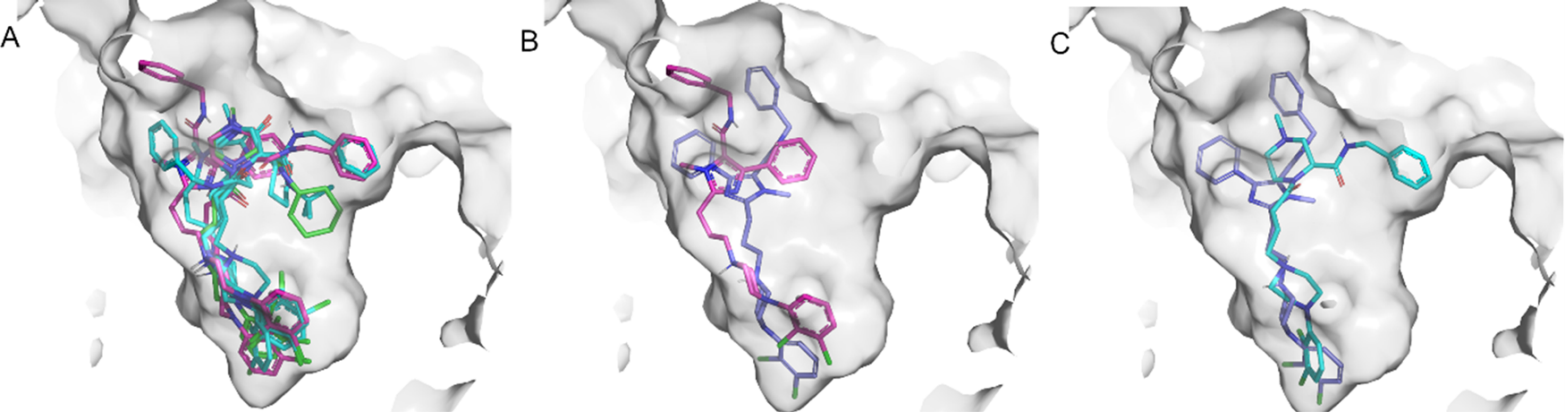
Binding mode of compounds in Table 2 on the active conformation of DRD_2_, PDB code 6CMS (A). Compounds are color coded according to their pharmacological activity as in Figure 5. The comparison with the corresponding binding pose on the inactive conformation of DRD_2_ is shown for compound **22a** (G-protein biased, B) and compound **27a** (β-arrestin biased, C).

During the course of this study, a structure of the active DRD_2_-G_i_ complex was revealed by cryo-EM.^65^ Despite the moderate resolution (3.8 Å), the binding mode of the orthosteric agonist bromocriptine is well evidenced. While mostly superimposing with risperidone, the bromine substituent does not reach as deep in the binding pocket as in the case of the fluorine of the antagonist, while in the EBD, the bicyclic tripeptide group of this agonist relatively overlays with the tetrahydropyridopyrimidone of risperidone.^65^ However, the differences in the conformation of the EL2 region between the two structures are notorious, favoring that the terminal part of the tripeptide in bromocriptine protrudes toward the extracellular tip of TM5. Consequently, an additional docking exploration of our compound series was performed using this active structure. Given the differences on the binding pocket of the head group, we had to impose additional flexibility during the docking exploration.^66^ The results, shown in Figure 6, reveal moderate adaptations of the binding pose of each compound as compared to the dockings on the inactive conformation of the receptor, mainly due to the impossibility to protrude as deep in the active conformation cavity as they do in the inactive conformation of the DRD_2_. Still, it is interesting that the G-protein biased ligand **22a** orients its benzyl tail toward the extracellular region of TM7, analogously to the inactive-bound configuration of this ligand (Figure 6B). In contrast, non-biased and β-arrestin biased ligands mostly show an alternative configuration of the SP substituents.

Altogether, the binding mode of each compound to both active and inactive conformations of the receptor suggest that the specific arrangement of the secondary pharmacophore (SP) substituent could be the key feature to the pharmacological profile of the ligands. Another interesting outcome is that, independent of the pharmacological nature of the ligand, only the (*R*) stereoisomers in series bearing a stereogenic center (**27**–**29**) can bind the receptor while maintaining the common interactions for the common parts of the ligands. Both of these aspects, i.e., the potential stereospecificity and the molecular mechanism associated to the ligand bias, are currently under further investigation in our groups.

## Conclusions

In summary, we have documented a previously unexplored multicomponent-based approach that enables the rapid generation of novel subtype-selective DRD_2_ biased ligands. This strategy exemplifies the search for diverse and previously unexplored structural elements binding the secondary pharmacophore but also highlights their critical role in modulating the functional selectivity profile. The pharmacological characterization of the new series of compounds enabled the identification of several ligands that elicit excellent DRD_2_ selectivity and remarkable functional selectivity by either the cAMP (**22a** and **29a**) or β-arrestin (**29c**, **27a** and **20b**) signaling pathways. These results can to some extent be explained by the molecular modeling of these ligands using the recent DRD_2_ experimental structures. Further studies are now in progress in our laboratory to expand the diversity of the PP, SP, and linkers, to explore in detail the SAR and SSR around ligands **22a**, **29a**, **29c**, and **27a**, and to establish the role of stereochemistry in the observed biological profiles.

## Experimental Section

### Chemistry

Unless stated otherwise, all starting materials, reagents and solvents were purchased and used without further purification. After extraction from aqueous phases, the organic solvents were dried over anhydrous sodium sulfate. The reactions were monitored by thin-layer chromatography (TLC) on 2.5 mm Merck silica gel GF 254 strips, and the purified compounds each showed a single spot, unless stated otherwise, UV light and/or iodine vapor were used for detection of compounds. The Ugi reactions were performed in coated Kimble vials on a PLS (6X4) Organic Synthesizer with orbital stirring. The purity and identity of all tested compounds were established by mass spectrometry, HRMS, and NMR spectra as described below. Purification of isolated products was carried out by column chromatography (Kieselgel 0.040–0.063 mm, E. Merck) or medium-pressure liquid chromatography (MPLC) on a CombiFlash Companion (Teledyne ISCO) with RediSep pre-packed normal-phase silica gel (35–60 μm) columns. Melting points were determined on a Gallenkamp melting point apparatus and are uncorrected. NMR spectra were recorded on Bruker AM300 and XM500 spectrometers. Chemical shifts are given as δ values against tetramethylsilane as an internal standard, and *J* values are given in Hz. Mass spectra were obtained on a Varian MAT-711 instrument. High-resolution mass spectra were obtained on an Autospec Micromass spectrometer. Routine purity control was performed by analytical HPLC using an Agilent 1100 system using an Agilent Zorbax SB-Phenyl, 2.1 mm × 150 mm, 5 μm column with gradient elution using the mobile phases (A) H_2_O containing 0.1% CF_3_COOH and (B) MeCN and a flow rate of 1 mL/min. The purity of all tested compounds was determined to be >95%. A detailed description of synthetic methodologies as well as analytical and spectroscopic data for all described compounds are provided below.

### General Procedure for the Synthesis of 4-(4-(2,3-Dichlorophenyl)piperazin-1-yl)-*N*-methylbutanamide Derivatives (**20a**–**c**)

A mixture of 4-(4-(2,3-dichlorophenyl)piperazin-1-yl)butanoic acid **16** (0.58 mmol), formaldehyde **18a** (0.58 mmol), methylamine **17a** (0.58 mmol), and the corresponding isocyanide (**19a–c**) (0.58 mmol, 1. eq) in methanol (2 mL) was stirred at 25 °C for 48 h. The reaction was monitored by TLC. After completion of the reaction, PS-*p*-TsOH (2.0 mmol) and CH_2_Cl_2_ (3 mL) were added. The reaction mixture was submitted to orbital stirring at room temperature until complete consumption of the unreacted isocyanide (30–60 min). The polystyrene-supported salt was filtered off and successively washed with MeOH (3 × 5 mL) and CH_2_Cl_2_ (3 × 5 mL). To the polystyrene-supported salt was added CH_2_Cl_2_ (3 mL) and DIPEA (3.0 eq), and the mixture was submitted to orbital stirring at room temperature for 60 min. Solvents were combined and evaporated to dryness. The resulting oil was purified by column chromatography on silica gel using MeOH/CH_2_Cl_2_.

#### *N*-(2-(Benzylamino)-2-oxoethyl)-4-(4-(2,3-dichlorophenyl)piperazin-1-yl)-*N*-methylbutanamide (**20a**)

Yield 64%. Mp: 88–90 °C. ^1^H NMR (300 MHz, CDCl_3_) δ (ppm): 7.36–7.20 (m, 5H), 7.19–7.08 (m, 2H), 6.99–6.88 (m, 1H), 6.67 (bs, 1H), 4.43 (d, *J* = 5.8 Hz, 2H), 4.04 (s, 2H), 3.14 (s, 3H), 3.04 (bs, 4H), 2.61 (bs, 4H), 2.49–2.38 (m, 4H), 1.98–1.75 (m, 2H). ^13^C NMR (75 MHz, CDCl_3_) δ (ppm): 174.1, 169.1, 151.3, 138.3, 134.2, 128.8, 128.8, 127.8, 127.8, 127.7, 127.7, 127.6, 124.7, 118.7, 57.7, 53.3, 53.3, 52.9, 51.3, 51.3, 43.5, 37.1, 30.9, 22.1. HRMS (CI) *m*/*z* calcd for C_24_H_31_Cl_2_N_4_O_2_ [M + H]^+^: 477.1824, found: 477.1840.

#### *N*-(2-(Cyclohexylamino)-2-oxoethyl)-4-(4-(2,3-dichlorophenyl)piperazin-1-yl)-*N*-methylbutanamide (**20b**)

Yield: 63%. Mp: 124–126 °C. ^1^H NMR (300 MHz, CDCl_3_) δ (ppm): 7.18–7.08 (m, 2H), 6.94 (dd, *J* = 6.3, 3.2 Hz, 1H), 6.20 (bs, 1H), 3.96 (s, 2H), 3.79–3.65 (m, 1H), 3.12 (s, 3H), 3.07 (s, 4H), 2.67 (bs, 4H), 2.52–2.41 (m, 4H), 1.94–1.80 (m, 4H), 1.76–1.52 (m, 2H), 1.46–1.04 (m, 6H). ^13^C NMR (75 MHz, CDCl_3_) δ (ppm): 173.9, 168.2, 151.3, 139.0, 134.2, 127.7, 124.8, 118.7, 57.7, 53.4, 53.3, 53.0, 51.3, 51.3, 48.2, 33.3, 33.1, 33.0, 30.9, 25.6, 24.8, 24.8, 22.1. HRMS (CI) *m*/*z* calcd for C_23_H_35_Cl_2_N_4_O_2_ [M + H]^+^: 469.2137, found: 469.2137.

#### *N*-(2-(*tert*-Butylamino)-2-oxoethyl)-4-(4-(2,3-dichlorophenyl)piperazin-1-yl)-*N*-methylbutanamide (**20c**)

Yield: 60%. Mp: 93–94 °C. ^1^H NMR (300 MHz, CDCl_3_) δ (ppm): 7.14–7.07 (m, 2H), 6.91 (dd, *J* = 5.9, 3.8 Hz, 1H), 6.04 (bs, 1H), 3.86 (s, 2H), 3.09 (s, 3H), 3.02 (bs, 4H), 2.61 (bs, 4H), 2.48–2.36 (m, 4H), 1.93–1.76 (m, 2H), 1.29 (s, 9H). ^13^C NMR (75 MHz, CDCl_3_) δ (ppm): 173.7, 168.3, 151.3, 134.0, 127.5, 127.5, 124.6, 118.7, 57.6, 53.4, 53.2, 51.3, 51.2, 36.9, 30.8, 28.8, 22.1. HRMS (CI) *m*/*z* calcd for C_21_H_33_Cl_2_N_4_O_2_ [M + H]^+^: 443.1981, found: 443.1984.

### General Procedure for the Synthesis of 2-(3-(4-(2,3-Dichlorophenyl)piperazin-1-yl)propyl)-1-methyl-4-phenyl-1*H*-imidazole-5-carboxamide Derivatives (**22a**–**c**)

A mixture of 4-(4-(2,3-dichlorophenyl)piperazin-1-yl)butanoic acid **16** (0.47 mmol), phenylglyoxal **18b** (0.47 mmol), methylamine **17a** (0.47 mmol), and the corresponding isocyanide (**19a–c**) (0.47 mmol) in trifluoroethanol (2 mL) was stirred at 45 °C for 48 h. The reaction was monitored by TLC. After completion of the reaction, PS-*p*-TsOH (2.0 mmol) and CH_2_Cl_2_ (3 mL) were added. The reaction mixture was submitted to orbital stirring at room temperature until complete consumption of the unreacted isocyanide (30–60 min). The polystyrene-supported salt was filtered off and successively washed with MeOH (3 × 5 mL) and CH_2_Cl_2_ (3 × 5 mL). To the polystyrene-supported salt was added CH_2_Cl_2_ (3 mL) and DIPEA (3.0 eq), and the mixture was submitted to orbital stirring at room temperature for 60 min. The solvents were combined and evaporated to dryness. The residue was dissolved in acetic acid (4 mL), NH_4_OAc (18.8 mmol, 40 eq) was added, and the reaction was stirred at 100 °C for 2–4 h. After completion of the reaction, the mixture was neutralized with a saturated aqueous NaHCO_3_ and extracted with AcOEt (2 × 10 mL) and CH_2_Cl_2_ (2 × 10 mL). The organic layers were combined and dried with Na_2_SO_4_, and the resulting oil was purified by column chromatography on silica gel using MeOH/CH_2_Cl_2_.

#### *N*-Benzyl-2-(3-(4-(2,3-dichlorophenyl)piperazin-1-yl)propyl)-1-methyl-4-phenyl-1*H*-imidazole-5-carboxamide (**22a**)

Yield: 35%. Mp: 145–147 °C. ^1^H NMR (300 MHz, CDCl_3_) δ (ppm): 7.51–7.42 (m, 2H), 7.32–7.22 (m, 6H), 7.19–7.07 (m, 4H), 6.94 (dd, *J* = 6.7, 2.9 Hz, 1H), 5.95 (t, *J* = 6.0 Hz, 1H), 4.42 (d, *J* = 5.9 Hz, 2H), 3.88 (s, 3H), 3.05 (bs, 4H), 2.81 (t, *J* = 7.6 Hz, 2H), 2.65 (bs, 4H), 2.54 (t, *J* = 7.1 Hz, 2H), 2.09–1.95 (m, 2H). ^13^C NMR (75 MHz, CDCl_3_) δ (ppm): 161.2, 151.1, 150.8, 142.3, 141.5, 137.4, 134.0, 134.0, 129.5, 129.1, 129.0, 128.9, 128.7, 128.6, 128.2, 128.1, 127.9, 127.5, 127.4, 124.7, 122.2, 118.7, 66.2, 57.5, 53.1, 51.1, 43.5, 32.3, 24.7, 24.6. HRMS (CI) *m*/*z* calcd for C_31_H_34_Cl_2_N_5_O [M + H]^+^: 562.2140, found: 562.2147.

#### *N*-Cyclohexyl-2-(3-(4-(2,3-dichlorophenyl)piperazin-1-yl)propyl)-1-methyl-4-phenyl-1*H*-imidazole-5-carboxamide (**22b**)

Yield: 30%. Mp: 140–142 °C. ^1^H NMR (300 MHz, CDCl_3_) δ (ppm): 7.59–7.52 (m, 2H), 7.44–7.32 (m, 3H), 7.17–7.12 (m, 2H), 6.95 (dd, *J* = 6.6, 3.0 Hz, 1H), 5.51 (d, *J* = 8.0 Hz, 1H), 3.89–3.78 (m, 4H), 3.06 (bs, 4H), 2.80 (t, *J* = 7.6 Hz, 2H), 2.67 (bs, 4H), 2.56 (t, *J* = 7.2 Hz, 2H), 2.10–1.94 (m, 2H), 1.86–1.71 (m, 2H), 1.60–1.47 (m, 3H), 1.40–1.21 (m, 2H), 1.16–0.80 (m, 3H). ^13^C NMR (75 MHz, CDCl_3_) δ (ppm): 160.4, 151.1, 150.4, 141.6, 134.1, 134.0, 129.2, 129.0, 128.6, 128.6, 128.2, 127.5, 124.6, 122.6, 118.6, 118.5, 57.5, 53.1, 51.1, 48.0, 32.4, 25.3, 24.8, 24.7, 24.5. HRMS (CI) *m*/*z* calcd for C_30_H_38_Cl_2_N_5_O [M + H]^+^: 554.2453, found: 554.2462.

#### *N*-(*tert*-Butyl)-2-(3-(4-(2,3-dichlorophenyl)piperazin-1-yl)propyl)-1-methyl-4-phenyl-1*H*-imidazole-5-carboxamide (**22c**)

Yield: 33%. Mp: 86–88 °C. ^1^H NMR (300 MHz, CDCl_3_) δ (ppm): 7.60–7.49 (m, 2H), 7.47–7.29 (m, 3H), 7.19–7.08 (m, 2H), 6.95 (dd, *J* = 6.6, 2.9 Hz, 1H), 5.44 (bs, 1H), 3.83 (s, 3H), 3.07 (bs, 4H), 2.80 (t, *J* = 7.5 Hz, 2H), 2.68 (bs, 4H), 2.62–2.50 (m, 2H), 2.05–1.99 (m, 2H), 1.21 (s, 9H). ^13^C NMR (75 MHz, CDCl_3_) δ (ppm): 160.8, 150.1, 134.2, 134.1, 132.1, 129.2, 128.7, 128.6, 128.2, 127.7, 127.5, 127.3, 126.1, 125.5, 118.9, 118.8, 76.8, 59.0, 52.9, 51.5, 49.8, 31.9, 28.5, 24.6, 22.7. HRMS (CI) *m*/*z* calcd for C_28_H_36_Cl_2_N_5_O [M + H]^+^: 528.2297, found: 528.2280.

### General Procedure for the Synthesis of 2-(2-(3-(4-(2,3-Dichlorophenyl)piperazin-1-yl)propyl)-1*H*-benzo[*d*]imidazol-1-yl)acetamide Derivatives (**24a**–**f**)

A mixture of 4-(4-(2,3-dichlorophenyl)piperazin-1-yl)butanoic acid **16** (0.47 mmol), formaldehyde **18a** (0.47 mmol), mono-BOC protected phenylenediamines **17b–c** (0.47 mmol), and the corresponding isocyanide (**19a–c**) (0.47 mmol) in methanol (2 mL) was stirred at 25 °C for 48 h. The reaction was monitored by TLC. After completion of the reaction, PS-*p*-TsOH (2.0 mmol) and CH_2_Cl_2_ (3 mL) were added. The reaction mixture was submitted to orbital stirring at room temperature until complete consumption of the unreacted isocyanide (30–60 min). The polystyrene-supported salt was filtered off and successively washed with MeOH (3 × 5 mL) and CH_2_Cl_2_ (3 × 5 mL). To the polystyrene-supported salt was added CH_2_Cl_2_ (3 mL) and DIPEA (1.41 mmol), and the mixture was submitted to orbital stirring at room temperature for 60 min. Solvents were combined and evaporated to dryness. The residue was dissolved in a 40% solution of trifluoroacetic acid in dichloroethane, and the reaction mixture was stirred at 85 °C for 3–4 h. After completion of the reaction, the mixture was neutralized with a saturated solution of NaHCO_3_, and the product was extracted with AcOEt (2 × 10 mL) and CH_2_Cl_2_ (2 × 10 mL). The organic layers were combined and dried with Na_2_SO_4_, and the resulting oil was purified by column chromatography on silica gel using MeOH/CH_2_Cl_2_.

#### *N*-Benzyl-2-(2-(3-(4-(2,3-dichlorophenyl)piperazin-1-yl)propyl)-1*H*-benzo[*d*]imidazol-1-yl)acetamide (**24a**)

Yield: 15%. Mp: 95–97 °C. ^1^H NMR (300 MHz, CDCl_3_) δ (ppm): 7.75–7.65 (m, 1H), 7.33–7.18 (m, 6H), 7.17–7.07 (m, 4H), 6.90 (dd, *J* = 6.5, 3.1 Hz, 1H), 6.03 (t, *J* = 5.7 Hz, 1H), 4.88 (s, 2H), 4.40 (d, *J* = 5.9 Hz, 2H), 2.98 (bs, 4H), 2.83 (t, *J* = 7.5 Hz, 2H), 2.58 (bs, 4H), 2.46 (t, *J* = 6.8 Hz, 2H), 2.13–1.93 (m, 2H). ^13^C NMR (75 MHz, CDCl_3_) δ (ppm): 166.7, 155.1, 151.1, 142.7, 137.3, 134.8, 134.0, 128.7, 127.7, 127.5, 124.7, 124.6, 123.2, 123.1, 122.9, 119.6, 118.7, 108.9, 57.2, 53.1, 51.1, 46.9, 43.4, 24.9, 24.4. HRMS (APCI) *m*/*z* calcd for C_29_H_32_Cl_2_N_5_O [M + H]^+**.**^: 536.1975, found: 536.1978.

#### *N*-Cyclohexyl-2-(2-(3-(4-(2,3-dichlorophenyl)piperazin-1-yl)propyl)-1*H*-benzo[*d*]-imidazol-1-yl)acetamide (**24b**)

Yield: 36%. Mp: 115–117 °C. ^1^H NMR (300 MHz, CDCl_3_) δ (ppm): 7.80–7.69 (m, 1H), 7.37–7.20 (m, 3H), 7.19–7.06 (m, 2H), 6.91 (dd, *J* = 6.6, 3.0 Hz, 1H), 5.25 (bs, 1H), 4.82 (s, 2H), 3.90–3.68 (m, 1H), 3.01 (bs, 4H), 2.90 (t, *J* = 6.5 Hz, 2H), 2.63 (bs, 4H), 2.54 (t, *J* = 6.8 Hz, 2H), 2.17–2.05 (m, 2H), 1.83–1.46 (m, 4H), 1.39–1.15 (m, 3H), 1.02–0.83 (m, 3H). ^13^C NMR (75 MHz, CDCl_3_) δ (ppm): 165.6, 155.2, 151.1, 142.6, 134.8, 134.0, 127.5, 124.6, 123.1, 122.8, 119.5, 119.4, 118.6, 109.0, 57.2, 53.1, 51.2, 48.6, 47.0, 32.7, 25.2, 24.9, 24.7, 24.5. HRMS (CI) *m*/*z* calcd for C_28_H_36_Cl_2_N_5_O [M + H]^+^: 528.2297, found: 528.2292.

#### *N*-(*tert*-Butyl)-2-(2-(3-(4-(2,3-dichlorophenyl)piperazin-1-yl)propyl)-1*H*-benzo[*d*]-imidazol-1-yl)acetamide (**24c**)

Yield: 30%. Mp: 91–93 °C. ^1^H NMR (300 MHz, CDCl_3_) δ (ppm): 7.79–7.70 (m, 1H), 7.33–7.23 (m, 3H), 7.18–7.09 (m, 2H), 6.91 (dd, *J* = 6.4, 3.2 Hz, 1H), 5.17 (bs, 1H), 4.73 (s, 2H), 3.01 (bs, 4H), 2.91 (t, *J* = 7.0 Hz, 2H), 2.63 (bs, 4H), 2.54 (t, *J* = 6.9 Hz, 2H), 2.18–2.04 (m, 2H), 1.24 (s, 9H). ^13^C NMR (75 MHz, CDCl_3_) δ (ppm): 165.8, 155.2, 151.2, 142.7, 134.8, 134.1, 127.6, 124.7, 123.1, 122.9, 119.7, 119.6, 118.7, 109.0, 57.4, 53.2, 52.0, 51.3, 47.6, 28.7, 25.1, 24.6. HRMS (CI) *m*/*z* calcd for C_26_H_34_Cl_2_N_5_O [M + H]^+^: 502.2140, found: 502.2127.

#### *N*-Benzyl-2-(5,6-dibromo-2-(3-(4-(2,3-dichlorophenyl)piperazin-1-yl)propyl)-1*H*-benzo[*d*]imidazol-1-yl)acetamide (**24d**)

Yield: 43%. Mp: 208–210 °C. ^1^H NMR (300 MHz, CDCl_3_) δ (ppm): 7.96 (s, 1H), 7.56 (s, 1H), 7.35–7.27 (m, 3H), 7.19–7.13 (m, 4H), 6.89 (dd, *J* = 6.6, 3.0 Hz, 1H), 5.86 (bs, 1H), 4.83 (s, 2H), 4.44 (d, *J* = 5.9 Hz, 2H), 2.99 (bs, 4H), 2.86 (t, *J* = 7.4 Hz, 2H), 2.61 (bs, 4H), 2.50 (t, *J* = 6.5 Hz, 2H), 2.15–1.98 (m, 2H). ^13^C NMR (75 MHz, DMSO-*d_6_*) δ (ppm): 166.4, 158.4, 151.2, 142.8, 138.9, 136.3, 132.6, 128.3, 127.3, 127.0, 126.0, 124.3, 122.6, 119.4, 115.6, 115.4, 114.8, 56.8, 52.7, 50.9, 45.8, 42.4, 24.4, 23.9. HRMS (CI) *m*/*z* calcd for C_29_H_30_Br_2_Cl_2_N_5_O [M + H]^+^: 692.0194, found: 692.0186.

#### *N*-Cyclohexyl-2-(5,6-dibromo-2-(3-(4-(2,3-dichlorophenyl)piperazin-1-yl)propyl)-1*H*-benzo[*d*]imidazol-1-yl)acetamide (**24e**)

Yield: 46%. Mp: 220–222 °C. ^1^H NMR (300 MHz, CDCl_3_) δ (ppm): 8.01 (s, 1H), 7.55 (s, 1H), 7.21–7.05 (m, 2H), 6.90 (dd, *J* = 6.3, 3.3 Hz, 1H), 5.22 (bs, 1H), 4.74 (s, 2H), 3.86–3.70 (m, 1H), 3.01 (bs, 4H), 2.89 (t, *J* = 7.4 Hz, 2H), 2.64 (bs, 4H), 2.55 (t, *J* = 6.7 Hz, 2H), 2.19–2.05 (m, 2H), 1.90–1.74 (m, 2H), 1.70–1.50 (m, 2H), 1.42–1.19 (m, 3H), 1.15–0.85 (m, 3H). ^13^C NMR (75 MHz, CDCl_3_) δ (ppm): 164.8, 157.4, 151.2, 143.2, 135.3, 134.2, 127.6, 124.8, 124.2, 118.7, 118.3, 118.2, 113.8, 107.4, 57.3, 53.3, 51.3, 49.0, 47.1, 33.0, 25.3, 25.1, 24.8, 24.4. HRMS (CI) *m*/*z* calcd for C_28_H_34_Br_2_Cl_2_N_5_O [M + H]^+^: 684.0507, found: 684.0494.

#### *N*-(*tert*-Butyl)-2-(5,6-dibromo-2-(3-(4-(2,3-dichlorophenyl)piperazin-1-yl)propyl)-1*H*-benzo[*d*]imidazol-1-yl)acetamide (**24f**)

Yield: 47%. Mp: 160–163 °C. ^1^H NMR (300 MHz, CDCl_3_) δ (ppm): 8.00 (s, 1H), 7.54 (s, 1H), 7.18–7.10 (m, 2H), 6.90 (dd, *J* = 6.3, 3.3 Hz, 1H), 5.20 (bs, 1H), 4.66 (s, 2H), 3.01 (bs, 4H), 2.89 (t, *J* = 7.4 Hz, 2H), 2.64 (bs, 4H), 2.55 (t, *J* = 6.8 Hz, 2H), 2.19–2.05 (m, 2H), 1.30 (s, 9H). ^13^C NMR (75 MHz, CDCl_3_) δ (ppm): 164.9, 157.4, 151.1, 142.8, 135.3, 134.1, 127.5, 124.8, 123.8, 123.7, 118.7, 117.9, 117.7, 113.6, 57.3, 53.2, 52.3, 51.1, 47.1, 28.7, 25.1, 24.2. HRMS (CI) *m*/*z* calcd for C_26_H_32_Br_2_Cl_2_N_5_O [M + H]^+^: 658.0351, found: 658.0334.

### General Procedure for the Synthesis of 4-(4-(4-(2,3-Dichlorophenyl)piperazin-1-yl)butanoyl)-3,4-dihydropyrazin-2(1*H*)-one Derivatives (**26a**–**c**)

A mixture of 4-(4-(2,3-dichlorophenyl)piperazin-1-yl)butanoic acid **16** (0.47 mmol), formaldehyde **18a** (0.47 mmol), aminoacetaldehyde dimethyl acetal **17d** (0.47 mmol), and the corresponding isocyanide (**19a–c**) (0.63 mmol) in methanol (2 mL) was stirred at 25 °C for 48 h. The reaction was monitored by TLC. After completion of the reaction, PS-*p*-TsOH (2.0 mmol) and CH_2_Cl_2_ (3 mL) were added. The reaction mixture was submitted to orbital stirring at room temperature until complete consumption of the unreacted isocyanide (30–60 min). The polystyrene-supported salt was filtered off and successively washed with MeOH (3 × 5 mL) and CH_2_Cl_2_ (3 × 5 mL). To the polystyrene-supported salt was added CH_2_Cl_2_ (3 mL) and DIPEA (1.41 mmol), and the mixture was submitted to orbital stirring at room temperature for 60 min. The polystyrene-supported salt was filtered off and successively washed with MeOH (3 × 5 mL) and CH_2_Cl_2_ (3 × 5 mL). The solutions were combined and evaporated to dryness. The residue was dissolved in a 50% solution of trifluoroacetic acid in dichloroethane, and the reaction mixture was stirred at 70 °C for 3 h. After the completion of the reaction, the mixture was neutralized with saturated aqueous NaHCO_3_ and the product was extracted with AcOEt (2 × 10 mL) and CH_2_Cl_2_ (2 × 10 mL). The organic layers were combined and dried with Na_2_SO_4_, and the resulting oil was purified by column chromatography on silica gel using MeOH/CH_2_Cl_2_.

#### 1-Benzyl-4-(4-(4-(2,3-dichlorophenyl)piperazin-1-yl)butanoyl)-3,4-dihydropyrazin-2(1*H*)-one (**26a**)

Yield: 15%. Mp: 120–122 °C.^1^H NMR (300 MHz, CDCl_3_) δ (ppm) (Mixture of rotamers): 7.37–7.17 (m, 5H), 7.16–7.04 (m, 2H), 6.90 (dd, *J* = 6.5, 3.2 Hz, 1H), 6.67 (d, *J* = 6.3 Hz, 0.3H), 6.20 (d, *J* = 6.1 Hz, 0.7H), 5.58 (d, *J* = 6.3 Hz, 0.3H), 5.53 (d, *J* = 6.1 Hz, 0.7H), 4.66 (s, 2H), 4.39 (s, 2H), 3.03 (bs, 4H), 2.69 (bs, 4H), 2.57–2.47 (m, 2H), 2.47–2.34 (m, 2H), 1.98–1.81 (m, 2H). ^13^C NMR (75 MHz, CDCl_3_) δ (ppm): 170.3, 163.4, 150.8, 135.9, 133.9, 128.8, 128.0, 127.5, 127.4, 124.7, 118.7, 112.7, 109.4, 57.2, 52.9, 50.8, 48.7, 45.9, 30.5, 21.2. HRMS (CI) *m*/*z* calcd for C_25_H_29_Cl_2_N_4_O_2_ [M + H]^+^: 487.1668, found: 487.1675.

#### 1-Cyclohexyl-4-(4-(4-(2,3-dichlorophenyl)piperazin-1-yl)butanoyl)-3,4-dihydropyrazin-2(1*H*)-one (**26b**)

Yield: 17%. Mp: 113–115 °C. ^1^H NMR (300 MHz, CDCl_3_) δ (ppm) (Mixture of rotamers): 7.19–7.05 (m, 2H), 6.93 (dd, *J* = 6.0, 3.6 Hz, 1H), 6.70 (d, *J* = 6.4 Hz, 0.3H), 6.24 (d, *J* = 6.2 Hz, 0.7H), 5.71 (d, *J* = 6.5 Hz, 0.3H), 5.65 (d, *J* = 6.2 Hz, 0.7H), 4.49–4.36 (m, 1H), 4.34 (s, 2H), 3.04 (bs, 4H), 2.62 (bs, 4H), 2.51–2.36 (m, 4H), 1.97–1.84 (m, 2H), 1.84–1.76 (m, 3H), 1.76–1.61 (m, 2H), 1.48–1.25 (m, 5H). ^13^C NMR (75 MHz, CDCl_3_) δ (ppm): 170.3, 162.9, 151.0, 134.0, 127.4, 124.6, 118.6, 109.3, 109.1, 109.0, 57.3, 53.1, 51.7, 51.0, 46.1, 30.8, 30.7, 25.5, 25.3, 21.5. HRMS (CI) *m*/*z* calcd for C_24_H_33_Cl_2_N_4_O_2_ [M + H]^+^: 479.1981, found: 479.1991.

#### 1-(*tert*-Butyl)-4-(4-(4-(2,3-dichlorophenyl)piperazin-1-yl)butanoyl)-3,4-dihydropyrazin-2(1*H*)-one (**26c**)

Yield: 15%. Mp: 139–141 °C. ^1^H NMR (300 MHz, CDCl_3_) δ (ppm) (Mixture of rotamers): 7.19–7.07 (m, 2H), 6.94 (dd, *J* = 6.0, 3.7 Hz, 1H), 6.58 (d, *J* = 6.5 Hz, 0.3H), 6.14 (d, *J* = 6.3 Hz, 0.7H), 5.94 (d, *J* = 6.6 Hz, 0.3H), 5.86 (d, *J* = 6.3 Hz, 0.7H), 4.26 (s, 2H), 3.04 (bs, 4H), 2.63 (bs, 4H), 2.53–2.34 (m, 4H), 2.01–1.81 (m, 2H), 1.47 (s, 9H). ^13^C NMR (75 MHz, CDCl_3_) δ (ppm): 170.1, 164.3, 151.1, 134.0, 127.5, 127.4, 124.7, 118.6, 111.6, 108.6, 58.3, 57.4, 53.1, 51.1, 47.5, 30.6, 28.5, 21.5. HRMS (CI) *m*/*z* calcd for C_22_H_31_Cl_2_N_4_O_2_ [M + H]^+^: 453.1824, found: 453.1805.

### General Procedure for the Synthesis of 1-(4-(4-(2,3-Dichlorophenyl)piperazin-1-yl)butanoyl)-4-methylpiperazine-2-carboxamide Derivatives (**27a**–**c**)

A mixture of 4-(4-(2,3-dichlorophenyl)piperazin-1-yl)butanoic acid **16** (0.63 mmol), 2-chloroacetaldehyde **18c** (0.63 mmol), *N*^1^-methylpropane-1,3-diamine **17e** (0.63 mmol), the corresponding isocyanide (**19a–c**) (0.63 mmol), and NaHCO_3_ (0.95 mmol) in trifluoroethanol (2 mL) was stirred at 25 °C for 72 h. The reaction was monitored by TLC. After completion of the reaction, PS-*p*-TsOH (2.0 mmol) and CH_2_Cl_2_ (3 mL) were added. The reaction mixture was submitted to orbital stirring at room temperature until complete consumption of the unreacted isocyanide (30–60 min). The polystyrene-supported salt was filtered off and successively washed with MeOH (3 × 5 mL) and CH_2_Cl_2_ (3 × 5 mL). To the polystyrene-supported salt was added CH_2_Cl_2_ (3 mL) and DIPEA (1.9 mmol, 3.0 equiv), and the mixture was submitted to orbital stirring at room temperature for 60 min. The polystyrene-supported salt was filtered off and successively washed with MeOH (3 × 5 mL) and CH_2_Cl_2_ (3 × 5 mL). Solvents were combined and evaporated to dryness. The resulting oil was purified by column chromatography on silica gel using MeOH/CH_2_Cl_2_.

#### (±) *N*-Benzyl-1-(4-(4-(2,3-dichlorophenyl)piperazin-1-yl)butanoyl)-4-methylpiperazine-2-carboxamide (**27a**)

Yield: 10%. Mp: 66–68 °C. ^1^H NMR (300 MHz, CDCl_3_) δ (ppm) (Mixture of rotamers): 8.00 (bs, 0.5H), 7.44–7.19 (m, 5H), 7.19–7.06 (m, 2H), 7.00–6.85 (m, 1H), 6.57 (bs, 0.5H), 5.26–5.12 (m, 0.5H), 4.60–4.36 (m, 2.5H), 3.81–3.68 (m, 0.5H), 3.51–3.35 (m, 1H), 3.30–3.18 (m, 0.5H), 3.11–2.98 (m, 4H), 2.95–2.75 (m, 2H), 2.71–2.58 (m, 5H), 2.51–2.41 (m, 3H), 2.30 (s, 1.5H), 2.28 (s, 1.5H), 2.10–1.77 (m, 4H). ^13^C NMR (75 MHz, CDCl_3_) δ (ppm): 172.8, 169.5, 138.1, 134.0, 128.9, 128.8, 128.7, 127.6, 127.5, 124.7, 124.6, 123.1, 118.6, 57.7, 55.6, 54.7, 54.4, 53.2, 51.2, 46.2, 43.5, 38.6, 30.8, 22.1. HRMS (CI) *m*/*z* calcd for C_27_H_36_Cl_2_N_5_O_2_ [M + H]^+^: 532.2246, found: 532.2246.

#### (±) *N*-Cyclohexyl-1-(4-(4-(2,3-dichlorophenyl)piperazin-1-yl)butanoyl)-4-methylpiperazine-2-carboxamide (**27b**)

Yield: 13%. Brown oil. ^1^H NMR (300 MHz, CDCl_3_) δ (ppm): 7.20–7.10 (m, 2H), 7.03–6.92 (m, 1H), 6.71 (bs, 1H), 4.43–4.25 (m, 1H), 3.81–3.70 (m, 1H), 3.49–3.30 (m, 1H), 3.27–3.02 (m, 5H), 2.85–2.48 (m, 8H), 2.30 (s, 3H), 2.08–1.77 (m, 6H), 1.48–1.04 (m, 10H). ^13^C NMR (75 MHz, CDCl_3_) δ (ppm): 169.5, 168.2, 149.0, 134.1, 127.6, 118.9, 118.8, 96.7, 57.6, 55.7, 53.1, 52.8, 45.9, 43.8, 40.7, 33.1, 33.0, 32.9, 32.8, 29.7, 25.5, 25.5, 24.8, 24.5, 22.7, 14.1. HRMS (CI) *m*/*z* calcd for C_26_H_40_Cl_2_N_5_O_2_ [M + H]^+^: 524.2559, found: 524.2560.

#### (±) *N*-(*tert*-Butyl)-1-(4-(4-(2,3-dichlorophenyl)piperazin-1-yl)butanoyl)-4-methylpiperazine-2-carboxamide (**27c**)

Yield: 10%. Brown oil. ^1^H NMR (300 MHz, CDCl_3_) δ (ppm) (Mixture of rotamers): 7.79 (bs, 0.6H), 7.20–7.05 (m, 2H), 6.95 (dd, *J* = 6.4, 3.3 Hz, 1H), 6.15 (bs, 0.4H), 5.07–4.96 (m, 0.4H), 4.57–4.42 (m, 0.6H), 4.37–4.22 (m, 0.6H), 3.79–3.66 (m, 0.4H), 3.48–3.36 (m, 0.4H), 3.36–3.26 (m, 0.6H), 3.18–2.97 (m, 4H), 2.97–2.74 (m, 2H), 2.72–2.59 (m, 4H), 2.59–2.34 (m, 4H), 2.28 (s, 3H), 2.24–2.09 (m, 1H), 2.07–1.77 (m, 3H), 1.33 (s, 9H). ^13^C NMR (75 MHz, CDCl_3_) δ (ppm): 172.8, 169.0, 168.7, 151.2, 134.1, 127.6, 124.8, 118.8, 57.8, 57.1, 55.8, 54.8, 54.6, 53.2, 53.0, 51.3, 51.0, 45.8, 38.4, 30.8, 29.0, 28.9, 28.8, 22.0. HRMS (CI) *m*/*z* calcd for C_24_H_38_Cl_2_N_5_O_2_ [M + H]^+^: 498.2403, found: 498.2404.

### General Procedure for the Synthesis of 1-(4-(4-(2,3-Dichlorophenyl)piperazin-1-yl)butanoyl)-3-oxopiperazine-2-carboxamide Derivatives (**29a**–**c**)

A mixture of 4-(4-(2,3-dichlorophenyl)piperazin-1-yl)butanoic acid **16** (0.63 mmol), ethyl glyoxylate **18d** (0.63 mmol), *N*-BOC-ethylenediamine **17f** (0.63 mmol), and the corresponding isocyanide (**19a–c**) (0.63 mmol) in methanol (2 mL) was stirred at 25 °C for 48 h. The reaction was monitored by TLC. After completion of the reaction, PS-*p*-TsOH (2.0 mmol) and CH_2_Cl_2_ (3 mL) were added. The reaction mixture was submitted to orbital stirring at room temperature until complete consumption of the unreacted isocyanide (30–60 min). The polystyrene-supported salt was filtered off and successively washed with MeOH (3 × 5 mL) and CH_2_Cl_2_ (3 × 5 mL). To the polystyrene-supported salt was added CH_2_Cl_2_ (3 mL) and DIPEA (1.9 mmol), and the mixture was submitted to orbital stirring at room temperature for 60 min. The polystyrene-supported salt was filtered off and successively washed with MeOH (3 × 5 mL) and CH_2_Cl_2_ (3 × 5 mL). The solutions were combined and evaporated to dryness. The residue was dissolved in a 10% solution of trifluoroacetic acid in dichloroethane, and the reaction mixture was stirred at 25 °C for 12 h. After completion of the reaction, the mixture was neutralized with a saturated solution of NaHCO_3_ and the product was extracted with AcOEt (2 × 10 mL) and CH_2_Cl_2_ (2 × 10 mL). The organic layers were combined and dried with Na_2_SO_4_, and the resulting oil was purified by column chromatography on silica gel using MeOH/CH_2_Cl_2_.

#### (±) *N*-Benzyl-1-(4-(4-(2,3-dichlorophenyl)piperazin-1-yl)butanoyl)-3-oxopiperazine-2-carboxamide (**29a**)

Yield: 19%. Mp: 160–162 °C. ^1^H NMR (300 MHz, CDCl_3_) δ (ppm) (Mixture of rotamers): 7.82 (t, *J* = 5.5 Hz, 0.3H), 7.42 (t, *J* = 5.5 Hz, 0.7H), 7.34–7.18 (m, 5H), 7.17–7.05 (m, 2H), 6.94 (dd, *J* = 6.4, 3.1 Hz, 1H), 6.92–6.79 (m, 1H), 5.51 (s, 0.7H), 5.19 (s, 0.3H), 4.80–4.67 (m, 0.3H), 4.59–4.31 (m, 2H), 4.03–3.86 (m, 1H), 3.71–3.54 (m, 0.7H), 3.50–3.31 (m, 2H), 3.04 (bs, 4H), 2.63 (bs, 4H), 2.53–2.34 (m, 2H), 2.34–2.16 (m, 2H), 2.01–1.74 (m, 2H). ^13^C NMR (75 MHz, CDCl_3_) δ (ppm): 172.1, 166.6, 164.8, 151.1, 137.8, 134.0, 128.7, 128.6, 127.6, 127.5, 124.6, 118.6, 61.4, 58.8, 57.4, 53.1, 51.0, 43.8, 41.1, 30.6, 21.7. HRMS (CI) *m*/*z* calcd for C_26_H_32_Cl_2_N_5_O_3_ [M + H]^+^: 532.1882, found: 532.1887.

#### (±) *N*-Cyclohexyl-1-(4-(4-(2,3-dichlorophenyl)piperazin-1-yl)butanoyl)-3-oxopiperazine-2-carboxamide (**29b**)

Yield: 11%. Mp: 92–94 °C. ^1^H NMR (300 MHz, CDCl_3_) δ (ppm) (Mixture of rotamers): 7.33 (d, *J* = 8.0 Hz, 0.6H), 7.19–7.06 (m, 2H), 6.93 (dt, *J* = 6.7, 3.5 Hz, 1H), 6.79 (d, *J* = 8.5 Hz, 0.4H), 6.68–6.48 (m, 1H), 5.46 (s, 0.6H), 5.08 (s, 0.4H), 4.80–4.65 (m, 0.4H), 4.03–3.91 (m, 0.6H), 3.81–3.54 (m, 2H), 3.53–3.22 (m, 2H), 3.05 (bs, 4H), 2.97–2.81 (m, 0.4H), 2.80–2.52 (m, 5H), 2.55–2.33 (m, 1.8H), 2.37–2.20 (m, 0.8H), 1.98–1.76 (m, 4H), 1.75–1.60 (m, 2H), 1.39–1.07 (m, 6H). ^13^C NMR (75 MHz, CDCl_3_) δ (ppm): 172.0, 166.8, 163.4, 151.1, 134.0, 127.5, 127.4, 124.6, 118.6, 58.7, 57.4, 53.1, 51.1, 48.9, 41.3, 40.8, 32.8, 32.7, 30.6, 25.4, 24.7, 24.6, 21.8. HRMS (CI) *m*/*z* calcd for C_25_H_36_Cl_2_N_5_O_3_ [M + H]^+^: 524.2195, found: 524.2189.

#### (±) *N*-(*tert*-Butyl)-1-(4-(4-(2,3-dichlorophenyl)piperazin-1-yl)butanoyl)-3-oxopiperazine-2-carboxamide (**29c**)

Yield: 12%. Mp: 164–166 °C. ^1^H NMR (300 MHz, CDCl_3_) δ (ppm) (Mixture of rotamers): 7.28 (s, 0.4H), 7.19–7.07 (m, 2H), 6.95 (dd, *J* = 6.3, 3.3 Hz, 1H), 6.82 (s, 0.6H), 6.30 (s, 0.6H), 6.26 (s, 0.4H), 5.43 (s, 0.6H), 5.01 (s, 0.4H), 4.84–4.68 (m, 0.4H), 4.04–3.85 (m, 1H), 3.69–3.55 (m, 0.6H), 3.55–3.23 (m, 2H), 3.07 (bs, 4H), 2.96–2.78 (m, 0.4H), 2.66 (bs, 4H), 2.60–2.38 (m, 3H), 2.32–2.20 (m, 0.6H), 2.02–1.81 (m, 2H), 1.36 (s, 3H), 1.34 (s, 6H). ^13^C NMR (75 MHz, CDCl_3_) δ (ppm): 171.9, 167.1, 163.2, 151.1, 134.0, 127.5, 127.4, 124.6, 118.6, 59.1, 57.5, 53.2, 53.1, 51.7, 51.1, 41.3, 40.7, 30.6, 28.6, 21.8. HRMS (CI) *m*/*z* calcd for C_23_H_34_Cl_2_N_5_O_3_ [M + H]^+^: 498.2039, found: 498.2029.

### Biological Evaluation

#### Cell Culture and Transient Transfection

HEK-293T cells were grown in Dulbecco’s modified medium (DMEM) (Gibco, Paisley, Scotland, UK) supplemented with 2 mM l-glutamine, 100 U/mL penicillin/streptomycin, MEM non-essential amino acids solution (1/100), and 5% (v/v) heat-inactivated fetal bovine serum (FBS) (Invitrogen, Paisley, Scotland, UK). Cells were maintained in a humid atmosphere of 5% CO_2_ at 37 °C. Cells were transiently transfected with the PEI (polyethyleneimine, Sigma-Aldrich) method as previously described.^67^

#### cAMP Determination

HEK-293T cells were transiently transfected with 0.5 μg of cDNA for DRD_2_, DRD_3_, or DRD_4_ with the PEI method. Two hours before initiating the experiment, the cell medium was exchanged to the non-supplemented DMEM medium. The cells were then detached and suspended in the medium containing 50 μM zardaverine. Cells were placed in 384-well plates (2500 cells/well), pretreated with antagonists or vehicle (15 min) and stimulated with agonists (15 min) before adding 0.5 μM forskolin or vehicle (15 min). Readings were performed after 1 h of incubation at 25 °C. Homogeneous time-resolved fluorescence energy transfer (HTRF) measurements were carried out using the Lance Ultra cAMP kit (Perkin Elmer, Waltham, MA, USA). Fluorescence at 665 nm was analyzed on a PHERAstar Flagship plate reader equipped with an HTRF optical module (BMG Lab Technologies, Offenburg, Germany). The reference value (100%) was that achieved by 0.5 μM forskolin treatment. The effect of ligands is given as a percentage with respect to the reference value.

#### β-Arrestin 2 Recruitment

HEK-293T cells were transiently transfected with 0.5 μg of cDNA β-arrestin 2-Rluc and with 0.6 μg of cDNA for DRD_2_-YFP, DRD_3_-YFP, or DRD_4_-YFP by the PEI method. Arrestin recruitment was determined as previously described.^57^ Briefly, BRET experiments were performed in HEK-293T cells 48 h after transfection with the cDNAs corresponding to the D_2_R-YFP and 1 μg of cDNA corresponding to β-arrestin 2-RLuc. Cells (20 μg of protein) were distributed in 96-well microplates (Corning 3600, white plates with white bottom) and were stimulated with the specific compounds for 10 min prior the addition of 5 μM coelenterazine H. Ten minutes after adding coelenterazine H, BRET between β-arrestin 2-RLuc and receptor-YFP was determined and quantified. The readings were collected using a Mithras LB-940 system (Berthold Technologies, Bad Wildbad, Germany), which allows the integration of the signals detected in the short-wavelengtsh filter at 485 nm and the long-wavelength filter at 530 nm. To quantify protein-RLuc expression, luminescence readings were also performed 10 min after adding 5 μM coelenterazine H.

#### Data Analysis

The data in graphs are the mean ± S.D. GraphPad Prism software version 5 (San Diego, CA, USA) was used for data fitting and statistical analysis. One-way ANOVA followed by a post-hoc Bonferroni test was used. Significant differences were considered when *p* < 0.05.

#### Molecular Modeling

The structure of hDRD_2_ in complex with the antagonist risperidone (PDB code 6CM4) and the active structure of the same receptor with agonist bromocriptine (PDB code 6VMS) were retrieved from the Protein Data Bank and prepared for docking purposes with the Protein Preparation Wizard from Maestro.^68^ This stage included addition of hydrogens, assignment of tautomeric states of His, Asn, and Gln sidechains, protonation state of ionizable residues considering physiological pH, as well as filling missing sidechains, replacing the fusion protein T4L with a hexapeptide based on the intracellular tips of TM5-TM6, and connecting these (as well as TM3-TM4) with Prime.^68^ The ligands with measured hDRD_2_ affinities (Table 1) were generated in their 3D conformation with Maestro from the corresponding smiles strings, and the database of ligands on the different protonation and tautomeric states generated with LigPrep. Three independent docking strategies were followed: (i) flexible ligand superposition within Maestro, using risperidone as a scaffold; (ii) automated docking with Glide, with the search box defined on the basis of the co-crystallized ligand (risperidone) and applying default single-precision (GlideSP) settings;^69^ (iii) induced-fit docking on the active structure with the IFD protocol implemented in the Schrödinger suite,^68^ with the search box defined on the basis of the co-crystallized ligand (bromocriptine). In each of these three strategies, one pose per ligand was retained, with separate stereoisomeric species considered when there was a stereocenter.

## Supporting Information Available

HPLC traces of lead compounds and HRMS (chemical ionization) of compounds **24d**, **24e**, and **24f** (PDF)
PDBs files (PDB, PDB)
Molecular formula strings (CSV)

## Abbreviations Used

7TMR: seven transmembrane receptors
APD: antipsychotic drug
DMEM: Dulbecco’s modified medium
DR: dopamine receptors
DRD_2_: dopamine D_2_ receptor
DRD_3_: dopamine D_3_ receptor
DRD_4_: dopamine D_3_ receptor
EBD: extended binding domain
EPS: extrapyramidal symptoms
GPCR: G protein-coupled receptor
HEK-293T: human embryonic kidney cell
LHS: left-hand side
MCR: multicomponent reaction
MOR: μ-opioid receptor
MPLC: medium-pressure liquid chromatography
PEI: polyethyleneimine
PLS: parallel reaction synthesizer
PP: primary pharmacophore
RHS: right-hand side
SD: standard deviation
SFSR: structure–functional selectivity relationship
SP: secondary pharmacophore
U-4CR: Ugi four-component reaction
UDC: Ugi-Deprotection-Cyclization
