# Supplementary material for: Potent and Subtype-Selective Dopamine D2 Receptor Biased Partial Agonists Discovered via an Ugi-Based Approach
Source: J Med Chem. 2021 Jun 10;64(12):8710–26. doi: 10.1021/acs.jmedchem.1c00704 (PMC8552448; doi:10.1021/acs.jmedchem.1c00704)
Supplement: Supplementary file 1 — jm1c00704_si_001.pdf [file jm1c00704_si_001.pdf]

SUPPORTING INFORMATION

Potent and Subtype-Selective dopamine D_2 receptor Biased partial agonists discovered via an Ugi-Based Approach

Ana Mallo-Abreu,[1,2] Irene Reyes-Resina,[3,5] Jhonny Azuaje,[1,2] Rafael Franco,[4,5] Aitor García-Rey,[1,2] Maria Majellaro,[1,2] Darío Miranda, [1,2] Xerardo García-Mera,[2] Willem Jespers,[6] Hugo Gutiérrez-de-Terán,[6] Gemma Navarro,[3,4*] and Eddy Sotelo[1,2*]

[1]Centro Singular de Investigación en Química Biolóxica e Materiais Moleculares (CIQUS), Universidade de Santiago de Compostela, 15782 Santiago de Compostela, Spain, and [2]Departamento de Química Orgánica, Facultade de Farmacia, Universidade de Santiago de Compostela, 15782 Santiago de Compostela, Spain. [3]Department of Biochemistry and Physiology, Faculty of Pharmacy and Food Science, University of Barcelona, 08028 Barcelona, Spain, [4]Faculty of Chemistry, University of Barcelona, 08028 Barcelona, Spain. [5]Centro de Investigación Biomédica en Red Enfermedades Neurodegenerativas (CIBERNED), 28031 Madrid, Spain. [6]Department of Cell and Molecular Biology, Uppsala University, Uppsala SE-75124.

TABLE OF CONTENTS

HPLC traces of lead compounds…………………………………………………….…S2

HRMS (chemical ionization) of compounds **24d**, **24e**, **24f**……………………….........S9

HPLC characterization of compounds 20b, 22a, 24d, 27a, 29a, and 29c. The purity determination was performed using a Water Breeze™ 2 (binary pump 1525, detector UV/Visible 2489, 7725i Manual Injector Kit 1500 Series) with a 150 mm x4.6 mm Luna® 5 µm Silica (2) 100Å (Phenomenex) using a linear gradient of mobile phase (DCM / *i*-propanol) at 25°C. All the compounds were detected at λ = 254 nm.

SAMPLE INFORMATION

Sample Name:	ISAM-A13	Acquired By:	Breeze
Sample Type:	Unknown	Sample Set Name:	
Vial:	1	Acq. Method:	10%IPA DCM
Injection #:	1	Date Acquired:	20/10/2020 15:34:24 CET
Run Time:	30,00 Minutes	Injection Volume:	20,00 ul

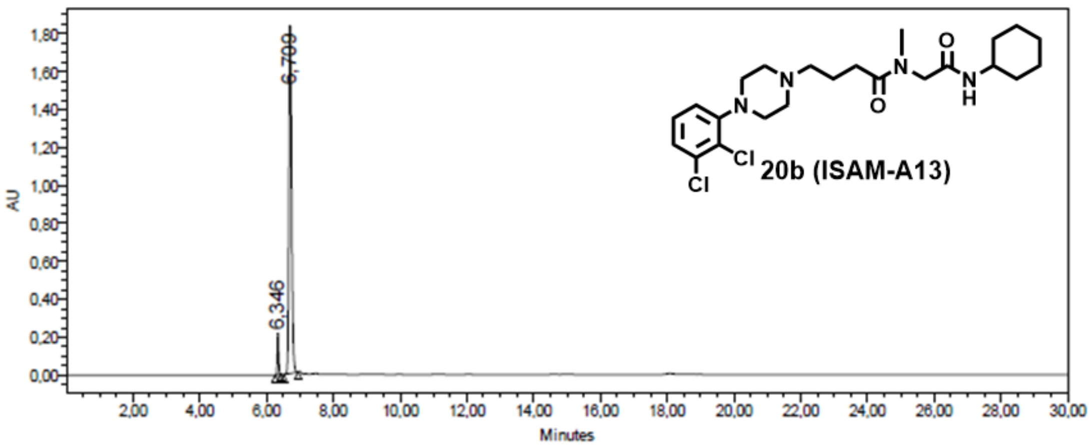

——— Channel: 2487Channel 1; Channel Desc.: ; Processing Method: 10%IPA DCM

	RT (min)	Area (µV*sec)	% Area	Height (µV)
1	6,346	616608	5,60	203995
2	6,709	10392455	94,40	1856371

Report Method: Injection Summary Report
Page: 1 of 1

Printed: 20/10/2020
19:19:05 Europe/Madrid

SAMPLE INFORMATION

Sample Name:	ISAM-A1684	Acquired By:	Breeze
Sample Type:	Unknown	Sample Set Name:	
Vial:	1	Acq. Method:	15%IPA DCM
Injection #:	3	Date Acquired:	20/10/2020 17:45:13 CET
Run Time:	30,00 Minutes	Injection Volume:	20,00 ul

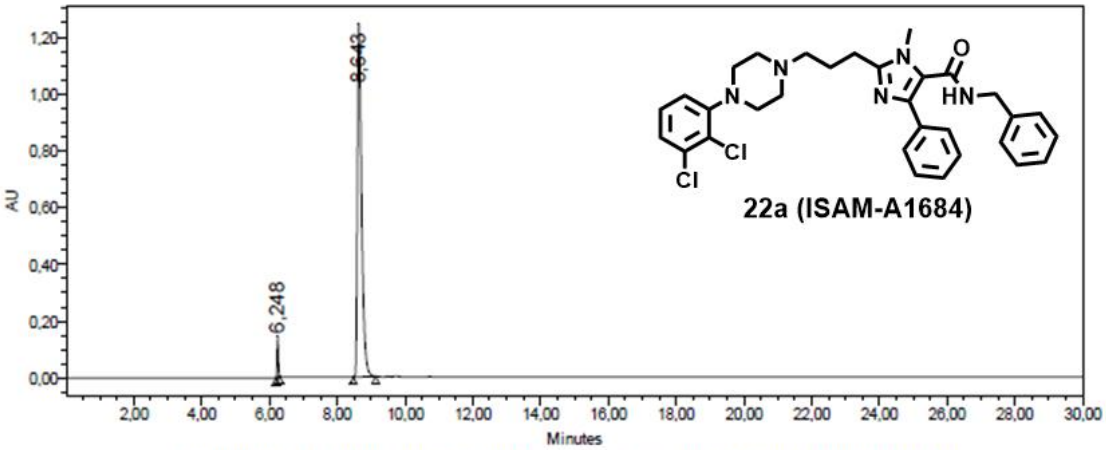

Channel: 2487Channel 1; Channel Desc.: ; Processing Method: 15%IPA DCM

	RT (min)	Area (µV*sec)	% Area	Height (µV)
1	6,248	361117	3,25	128694
2	8,643	10733607	96,75	1267603

Report Method: Injection Summary Report
Page: 1 of 1

Printed: 20/10/2020
19:41:13 Europe/Madrid

SAMPLE INFORMATION

Sample Name:	ISAM-A18	Acquired By:	Breeze
Sample Type:	Unknown	Sample Set Name:	
Vial:	1	Acq. Method:	10% IPA DCM
Injection #:	1	Date Acquired:	21/10/2020 16:24:42 CEST
Run Time:	30,00 Minutes	Injection Volume:	20,00 ul

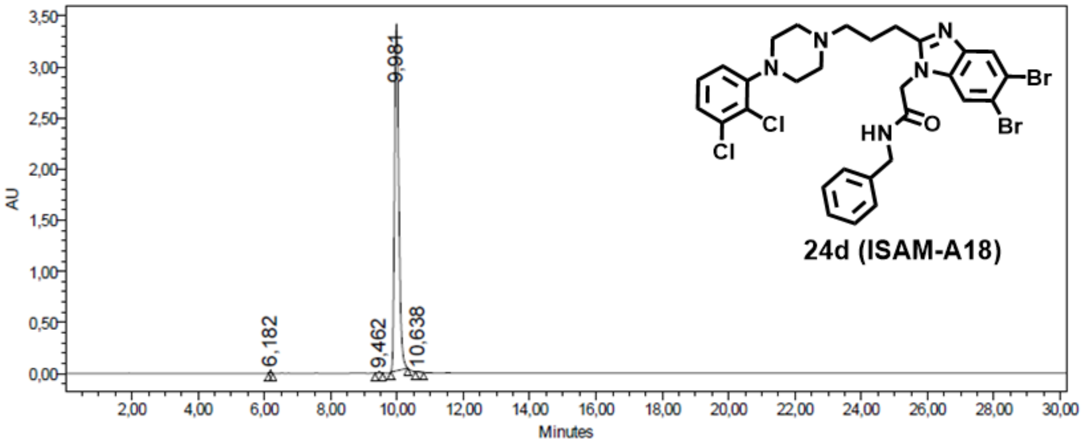

Channel: 2487Channel 1; Channel Desc.: ; Processing Method: 10% IPA DCM

	Channel Description	RT (min)	Area (µV*sec)	% Area	Height (µV)
1	254	6,182	102273	0,33	32474
2	254	9,462	74852	0,24	11106
3	254	9,981	30515466	99,30	3398223
4	254	10,638	38041	0,12	4964

Report Method: Injection Summary Report
Page: 1 of 1

Printed: 21/10/2020
17:05:26 Europe/Madrid

SAMPLE INFORMATION

Sample Name:	ISAM-A51	Acquired By:	Breeze
Sample Type:	Unknown	Sample Set Name:	
Vial:	1	Acq. Method:	15%IPA DCM
Injection #:	1	Date Acquired:	21/10/2020 14:01:27 CET
Run Time:	30,00 Minutes	Injection Volume:	20,00 ul

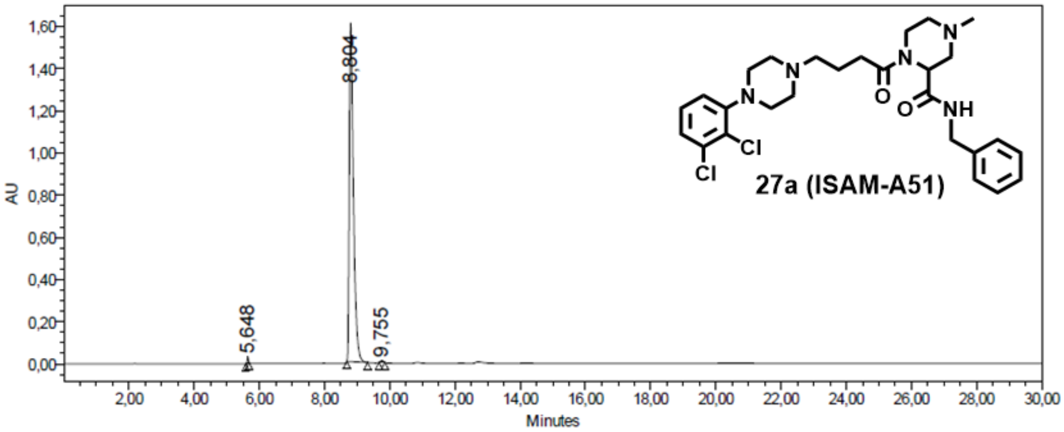

Channel: 2487Channel 1; Channel Desc.: ; Processing Method: 15%IPA DCM

	RT (min)	Area (µV*sec)	% Area	Height (µV)
1	5,648	70831	0,50	25673
2	8,804	14113870	99,14	1608696
3	9,755	51148	0,36	8169

Report Method: Injection Summary Report
Page: 1 of 1

Printed: 21/10/2020
14:32:50 Europe/Madrid

SAMPLE INFORMATION

Sample Name:	ISAM-A53	Acquired By:	Breeze
Sample Type:	Unknown	Sample Set Name:	
Vial:	1	Acq. Method:	15%IPA DCM
Injection #:	2	Date Acquired:	20/10/2020 16:36:09 CET
Run Time:	30,00 Minutes	Injection Volume:	20,00 ul

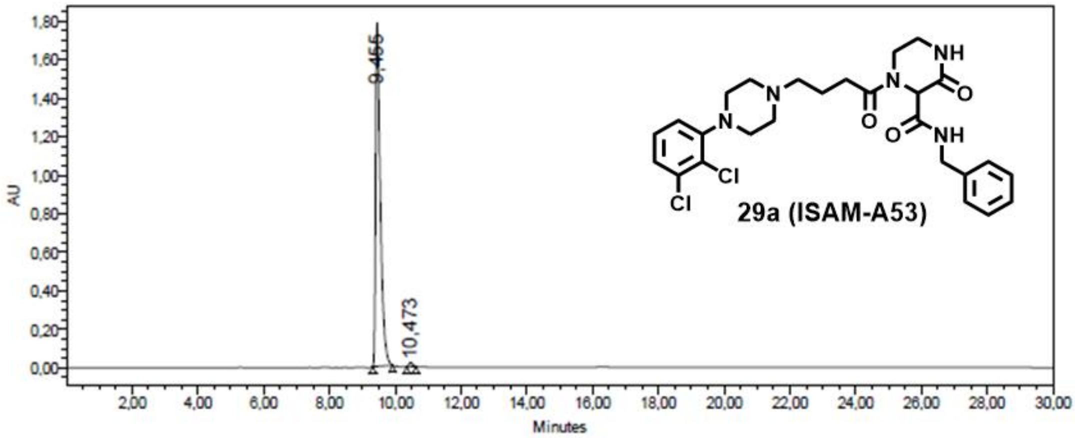

Channel: 2487Channel 1; Channel Desc.: ; Processing Method: 15%IPA DCM

	RT (min)	Area (µV*sec)	% Area	Height (µV)
1	9,455	17624197	98,99	1791568
2	10,473	179898	1,01	21993

Report Method: Injection Summary Report
Page: 1 of 1

Printed: 20/10/2020
19:26:27 Europe/Madrid

SAMPLE INFORMATION

Sample Name:	ISAM-A52	Acquired By:	Breeze
Sample Type:	Unknown	Sample Set Name:	
Vial:	1	Acq. Method:	15% IPA DCM
Injection #:	1	Date Acquired:	21/10/2020 16:12:43 CEST
Run Time:	30,00 Minutes	Injection Volume:	20,00 ul

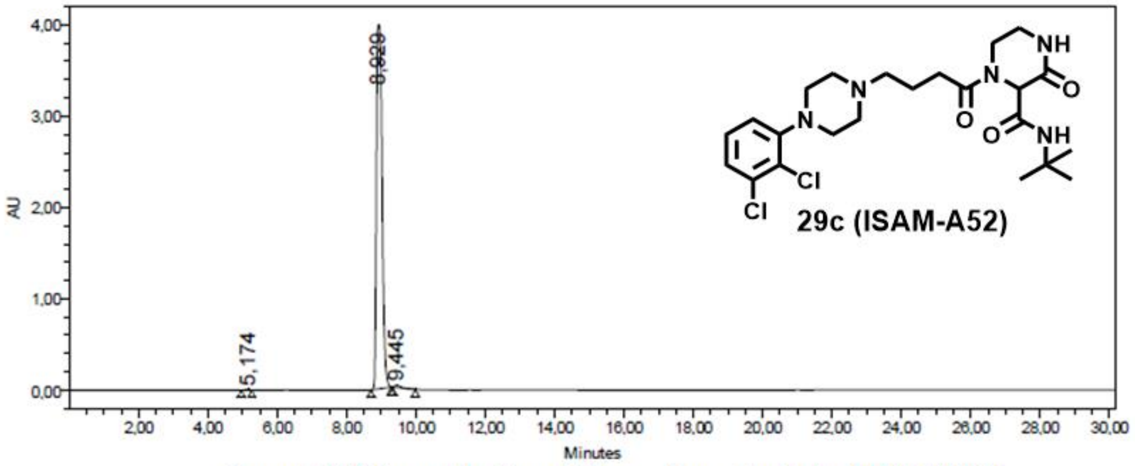

Channel: 2487Channel 1; Channel Desc.: ; Processing Method:15%IPA DCM

	Channel Description	RT (min)	Area (µV*sec)	% Area	Height (µV)
1	254	5,174	73675	0,16	10135
2	254	8,929	44673839	99,22	4028253
3	254	9,445	279446	0,62	25001

Elemental Composition Report

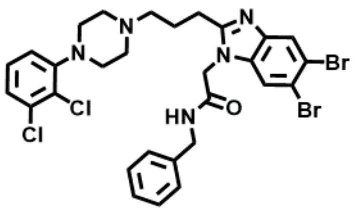

24d (ISAM-A18)

Multiple Mass Analysis: 18 mass(es) processed - displaying only valid results
Tolerance = 50.0 PPM / DBE: min = -1.5, max = 90.0
Selected filters: None

Monoisotopic Mass, Odd and Even Electron Ions
1 formula(e) evaluated with 1 results within limits (up to 50 best isotopic matches for each mass)
Elements Used:
C: 29-29 H: 30-30 N: 5-5 O: 1-1 Cl: 2-2 Br: 2-2

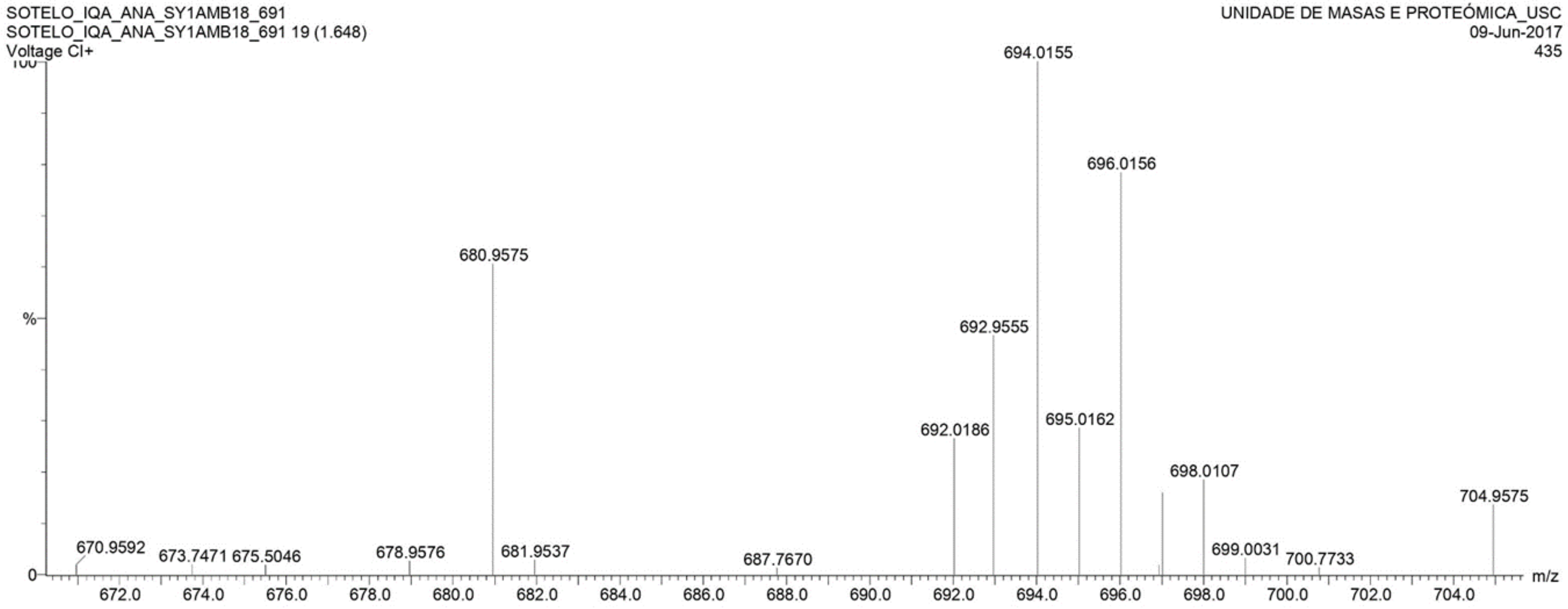

Minimum:	1.00				-1.5		
Maximum:	100.00		5.0	50.0	90.0		
Mass	RA	Calc. Mass	mDa	PPM	DBE	i-FIT	Formula
692.0186	26.45	692.0194	-0.8	-1.2	15.5	67.7	C29 H30 N5 O Cl2 Br2

Elemental Composition Report

Multiple Mass Analysis: 12 mass(es) processed - displaying only valid results
Tolerance = 50.0 PPM / DBE: min = -1.5, max = 90.0
Selected filters: None

Monoisotopic Mass, Odd and Even Electron Ions
1 formula(e) evaluated with 1 results within limits (up to 50 best isotopic matches for each mass)
Elements Used:
C: 28-28 H: 34-34 N: 5-5 O: 1-1 Cl: 2-2 Br: 2-2

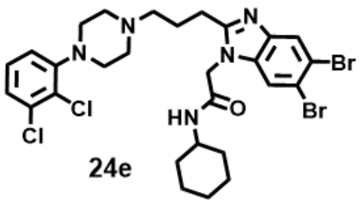

24e

SOTELO_IQA_ANA_SY1AMB16_683
SOTELO_IQA_ANA_SY1AMB16_683 19 (1.648)
Voltage CI+

UNIDADE DE MASAS E PROTEÓMICA_USC
09-Jun-2017
2.09e4

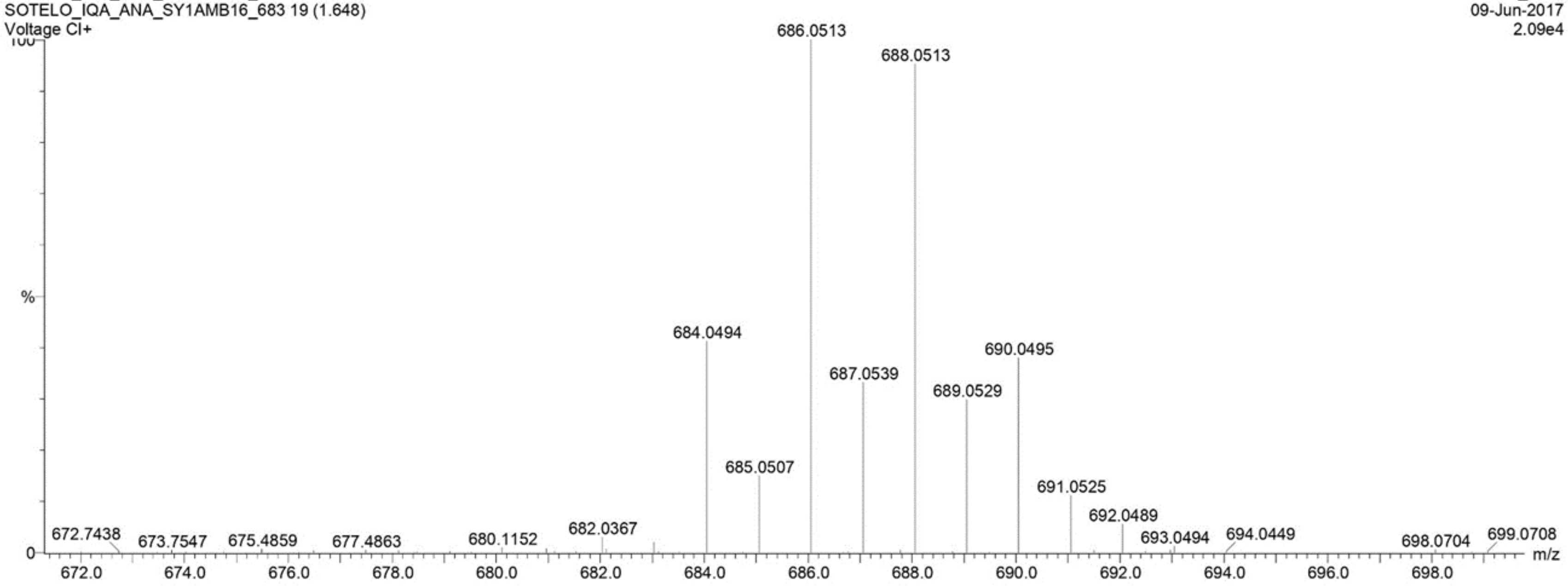

Minimum:	1.00				-1.5		
Maximum:	100.00		5.0	50.0	90.0		
Mass	RA	Calc. Mass	mDa	PPM	DBE	i-FIT	Formula
684.0494	41.14	684.0507	-1.3	-1.9	12.5	44.5	C28 H34 N5 O Cl2 Br2

Elemental Composition Report

Multiple Mass Analysis: 23 mass(es) processed - displaying only valid results
Tolerance = 50.0 PPM / DBE: min = -1.5, max = 90.0
Selected filters: None

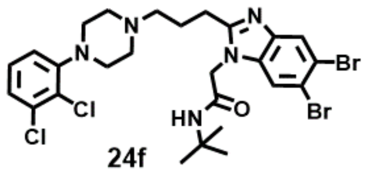

24f

Monoisotopic Mass, Odd and Even Electron Ions
1 formula(e) evaluated with 1 results within limits (up to 50 best isotopic matches for each mass)
Elements Used:
C: 26-26 H: 32-32 N: 5-5 O: 1-1 Cl: 2-2 Br: 2-2

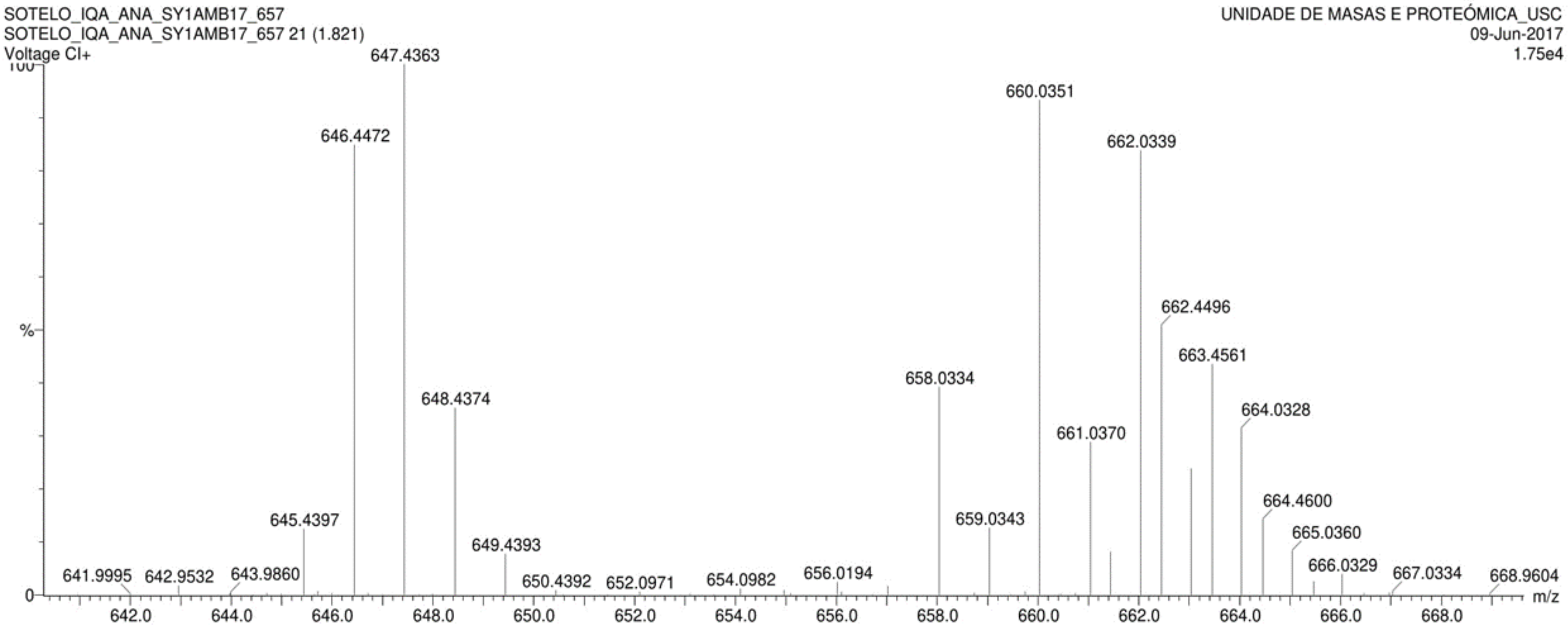

Minimum:	1.00				-1.5		
Maximum:	100.00		5.0	50.0	90.0		
Mass	RA	Calc. Mass	mDa	PPM	DBE	i-FIT	Formula
658.0334	39.16	658.0351	-1.7	-2.6	11.5	33.8	C26 H32 N5 O Cl2 Br2
